# Single atom catalysts in Van der Waals gaps

**DOI:** 10.1038/s41467-022-34572-3

**Published:** 2022-11-11

**Authors:** Huaning Jiang, Weiwei Yang, Mingquan Xu, Erqing Wang, Yi Wei, Wei Liu, Xiaokang Gu, Lixuan Liu, Qian Chen, Pengbo Zhai, Xiaolong Zou, Pulickel M. Ajayan, Wu Zhou, Yongji Gong

**Affiliations:** 1 School of Materials Science and Engineering, Beihang University, Beijing, 100191 China; 2 School of Materials and Chemistry, University of Shanghai for Science and Technology, 200093 Shanghai, P.R. China; 3 School of Physical Sciences and CAS Key Laboratory of Vacuum Physics, University of Chinese Academy of Sciences, Beijing, 100049 China; 4 Shenzhen Geim Graphene Center and Low-Dimensional Materials and Devices Laboratory, Tsinghua-Berkeley Shenzhen Institute, Tsinghua University, Shenzhen, 518055 China; 5 State Key Laboratory of Organic-Inorganic Composites, Beijing Key Laboratory of Electrochemical Process and Technology for Materials, Beijing University of Chemical Technology, Beijing, 100029 China; 6 College of Physics, Qingdao University, Qingdao, 266071 China; 7 School of Material Science & NanoEngineering, Rice University, Houston, TX 77005 USA; 8 Center for Micro-Nano Innovation of Beihang University, Beijing, 100191 China

**Keywords:** Electrocatalysis, Two-dimensional materials, Solid-state chemistry

## Abstract

Single-atom catalysts provide efficiently utilized active sites to improve catalytic activities while improving the stability and enhancing the activities to the level of their bulk metallic counterparts are grand challenges. Herein, we demonstrate a family of single-atom catalysts with different interaction types by confining metal single atoms into the van der Waals gap of two-dimensional SnS_2_. The relatively weak bonding between the noble metal single atoms and the host endows the single atoms with more intrinsic catalytic activity compared to the ones with strong chemical bonding, while the protection offered by the layered material leads to ultrahigh stability compared to the physically adsorbed single-atom catalysts on the surface. Specifically, the trace Pt-intercalated SnS_2_ catalyst has superior long-term durability and comparable performance to that of commercial 10 wt% Pt/C catalyst in hydrogen evolution reaction. This work opens an avenue to explore high-performance intercalated single-atom electrocatalysts within various two-dimensional materials.

## Abstract (web summary)

A family of single-atom catalysts synthesized by intercalating metal single atoms into the van der Waals gap of two-dimensional SnS_2_ is reported. The materials are applied as hydrogen evolving catalysts with good durability and overpotential.

## Introduction

Single-atom catalysts (SACs) are an emerging class of catalysts with maximized atom utilization efficiency and display excellent catalytic activities for many important reactions^1–13^. In order to achieve atomic dispersion of the supported metal species, some metal oxides^14^, carbon-based materials^15,16^, porous materials^17–19^, and two-dimensional (2D) materials^20,21^ have been employed to anchor metal single atoms. Nevertheless, metal atoms on such hosts still suffer high oxidation states^22^ or insufficient stability under reaction conditions^22–25^.

Recently, a solution-based intercalation method has been developed to introduce metal atoms into the van der Waals (vdW) gaps of 2D layered materials, where the loading-controllable intercalated atoms are uniformly distributed in the 2D limit, resulting in dramatically tuned electronic properties and striking stability^26^. The feasibility of this intercalation method promotes the diversity of host materials and guest metal atoms^27–29^. Noticeably, some intercalated metal atoms usually interact relatively weakly with the supporting 2D materials via unsaturated charge transfer, which might confer the metal single atoms similar intrinsic properties to bulk states due to their comparatively low oxidation states and make the guest materials more active^26,30–33^. In addition, the intercalated metal atoms, confined in the vdW gap, rather than physically adsorbed or anchored on the surface, are likely to possess high structural stability under the protection of the layered hosts. Therefore, direct intercalation of metal atoms, especially noble metal atoms into the vdW gaps of 2D materials might be a reasonable approach to construct a supplemental family of SACs with enhanced catalytic performance and stability.

In this work, we report a family of SACs with different interaction types by intercalating different metal single atoms into the vdW gap of SnS_2_ on reduced graphene oxide (rGO, denoted as Cu, Ni, Pd, and Pt-SnS_2_/rGO). Hydrogen evolution reaction (HER) is used as a model reaction to explore the potential of metal-intercalation in electrocatalysis. We show that Pt-SnS_2_/rGO with only ~1 wt% Pt loading shows superior HER performance, including similar overpotential, lower Tafel slope, and superior long-term stability (over 50,000 cycles) compared to commercial 10 wt% Pt/C catalyst. Scanning transmission electron microscopy (STEM) and X-ray absorption fine structure (XAFS) analysis unambiguously substantiate the atomic dispersion of Pt atoms in the vdW gap of the SnS_2_ nanosheets. X-ray photoelectron spectroscopy (XPS) and XAFS analysis both manifest the relatively weak bonding between Pt atoms and SnS_2_/rGO. Density functional theory (DFT) calculations confirm the structure stability and catalytic activity of Pt-SnS_2_/rGO system and reveal that the Pt single atoms in the vdW gap have an ultralow Gibbs free energy (|Δ*G*_H*_|) for hydrogen adsorption (0.01 eV), reaching the intrinsic activity of Pt metal. This universal intercalation method provides a strategy to construct highly efficient and stable single-atom electrocatalysts confined within the vdW gaps of various 2D materials.

## Results

### Feasibility of intercalation

**Fig. 1:**
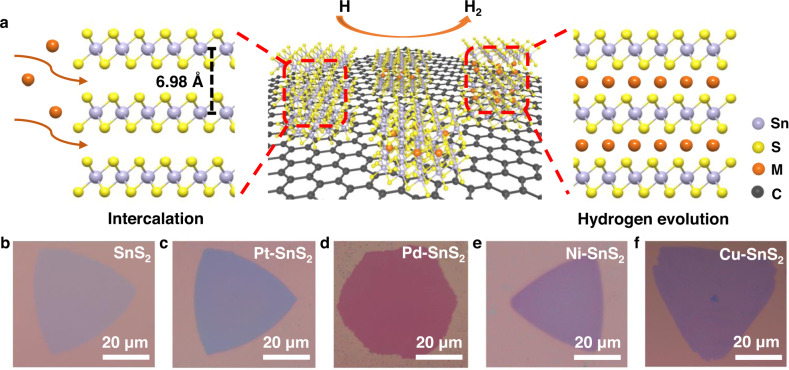
Realization of Pt-, Pd-, Ni-, and Cu-SnS_2_ through a solution-based intercalation method. **a** Schematic illustration of the intercalation of layered SnS_2_. **b**–**f** Optical images of pristine chemical vapor deposition (CVD) grown SnS_2_ (**b**) and CVD-grown SnS_2_ after intercalation of various metal atoms: **c** Pt-SnS_2_. **d** Pd-SnS_2_. **e** Ni-SnS_2_. **f** Cu-SnS_2_.

Since the color change of ultrathin SnS_2_ on Si/SiO_2_ substrate is a strong indication of the intercalation reaction, chemical vapor deposition (CVD) grown SnS_2_ was used to verify the feasibility of the solution-based intercalation method to introduce various transition metals into the vdW gap^26^. With a relatively slow rate for dispersing the metal atoms in the solution under appropriate concentration and temperature, successful intercalation could be uniformly realized (see Methods in Supplementary Materials for details). Figure 1a shows the schematics for the intercalation process. The drastic color change in the optical images before (Fig. 1b) and after (Fig. 1c–f) the intercalation indicates that the electronic structure and optical properties of SnS_2_ could be successfully tuned by intercalation of different metal atoms (Pt, Pd, Ni, and Cu), see Supplementary Fig. 1 for more detailed explanations. The change of band structure after intercalation can be confirmed by changed device performances as shown in Supplementary Fig. 2. The loading of the guest metal atoms in the vdW gap could be tuned by controlling the solution concentration or the intercalation time. Despite the small intercalated metal concentration, the intercalated atoms can be homogeneously distributed when the reaction temperature, time, and concentration are appropriate, while too short time results in uneven intercalation (Supplementary Fig. 3). Two Pt-SnS_2_ samples in deep pink and purple colors respectively were obtained by intercalating different amounts of Pt into SnS_2_ (Supplementary Fig. 4). No obvious change to the sample morphology was observed after intercalation, demonstrating that the intercalation process does not cause structural degradation (Supplementary Fig. 5). The morphology and the color of the SA-intercalated SnS_2_ samples remained stable after exposure to air for several months (Supplementary Fig. 6), which is beneficial for practical applications.

The successful intercalation of these SnS_2_ nanosheets was further confirmed by Raman spectroscopy analysis (Supplementary Fig. 7). Pristine SnS_2_ shows only one Raman peak at 313.6 cm^−1^ corresponding to the out-plane vibration mode (*A*_1g_)^34^, whereas this peak splits into two main peaks for the intercalated SnS_2_, similar to the intercalation of FeCl_3_ to multi-layer graphene^35,36^. On the one hand, the split of the characteristic Raman peak is attributed to the intercalation of SA metal to layered SnS_2_. On the other hand, the low-wavenumber peaks for the four intercalated SnS_2_ all exhibit redshifts from the original SnS_2_ characteristic peak, indicating weak interlayer coupling between adjacent SnS_2_ layers after intercalation. Further, the reproducible Raman spectra obtained at different plots of the four intercalated SnS_2_ samples (Supplementary Fig. 8) substantiate the homogeneous distribution of intercalation, consistent with the uniform colors in the OM images of the intercalated nanosheets under same thicknesses (Supplementary Fig. 9).

To further corroborate the successful intercalation of metal atoms into the vdW gap of SnS_2_, cross-sectional scanning transmission electron microscopy high-angle annular dark-field (STEM-HAADF) imaging at the atomic scale was performed for the Pt-intercalated SnS_2_ on a silicon substrate (Supplementary Fig. 10). The enlarged STEM-HAADF image in Supplementary Fig. 10b exhibits clear contrast features of Pt atoms in the vdW gap of SnS_2_, and the corresponding line intensity profiles (Supplementary Fig. 10c) provide solid evidence for the presence of single Pt atoms in the vdW gap. The Sn-Pt-Sn distance is merely 2.5% higher than Sn-Sn distance, in accord with some intercalated materials by similar methods^27,28,30,37^ and intrinsic intercalated materials such as M_1/3_NS_2_ (M = Mn, Fe, Co, Ni; N = Nb, Ta. Supplementary Table 1). The high-resolution transmission electron microscopy (HRTEM) image and the corresponding fast Fourier transform (FFT) pattern show only one set of crystal lattice of SnS_2_ phase in the Pt-intercalated SnS_2_ sample (Supplementary Fig. 11), excluding the formation of Pt nanoparticles on the surface. The atomic-scale electron microscopy results prove that Pt atoms had been successfully intercalated into the interlayer gap of vdW SnS_2_ crystal using our simple solution-based method.

### Structural and compositional characterization

Considering the unique atomic distribution of intercalators between SnS_2_ layers and the remarkable diversity of intercalated metal atoms in SnS_2_, especially noble metal atoms (Pt and Pd), intercalated SnS_2_ might be conducted as a supplemental kind of SACs. To make full use of the metal atoms in the vdW gap as catalysts, we further fabricated SnS_2_/rGO hybrid nanosheets to provide a 3D host for the subsequent metal atoms intercalation process (see Methods in Supplementary Materials for details)^35^. The SnS_2_ nanoflakes on rGO support have a homogeneous size distribution of ~4 nm in diameter (Supplementary Fig. 12). In contrast, the same synthesis process without rGO support produced SnS_2_ nanoplates with the thickness of tens of nanometers and the lateral size in the micrometer scale (Supplementary Fig. 13). The downsized SnS_2_ nanoflakes on rGO could further shorten the diffusion distance of metal atoms and protons during the intercalation reaction and the catalytic reaction, respectively. Thus, the use of rGO is necessary, as it helps to decrease the size of SnS_2_ nanoflakes and works as the conductive backbone.

**Fig. 2:**
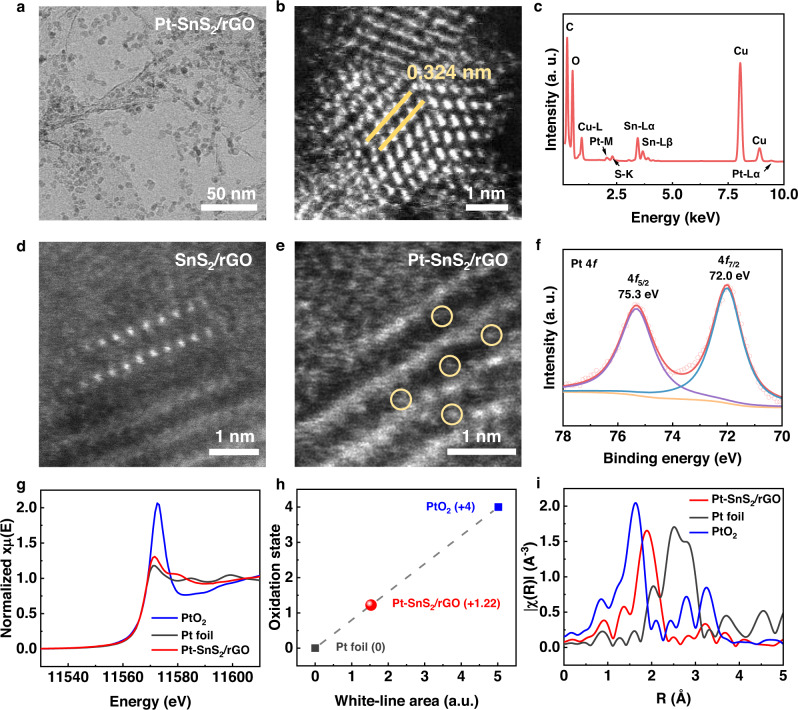
Structural characterization. **a** Bright-field transmission electron microscopy (TEM) image of Pt-SnS_2_/rGO. **b** Scanning transmission electron microscopy high-angle annular dark-field (STEM-HAADF) image of Pt-SnS_2_/rGO. **c** The energy-dispersive X-ray spectroscopy (EDS) spectrum of Pt-SnS_2_/rGO. **d** STEM-HAADF image of vertical SnS_2_. **e** STEM-HAADF image of Pt-intercalated vertical SnS_2_. **f** X-ray photoelectron spectroscopy (XPS) core-level spectrum of Pt 4 *f* for Pt-SnS_2_/rGO. **g** The normalized X-ray absorption near-edge structure (XANES) at the Pt L_3_-edge of Pt-SnS_2_/rGO, Pt foil, and PtO_2_. **h** The fitting of Pt oxidation state for Pt-SnS_2_/rGO, Pt foil, and PtO_2_ according to the white-line area of XANES. **i** Fourier transform extended X-ray absorption fine structure (FT-EXAFS) region for the local coordination structures of Pt in Pt-SnS_2_/rGO Pt foil, and PtO_2_.

The as-prepared SnS_2_/rGO hybrid nanosheets were subsequently intercalated with various metal atoms (Pt, Pd, Ni, and Cu) in solution. The typical amount of intercalated Pt in the Pt-SnS_2_/rGO catalysts was ~1.0 wt%, as measured by inductively coupled plasma-mass spectrometry (ICP-MS). TEM and scanning electron microscope (SEM) images of the 1.0 wt% Pt-SnS_2_/rGO sample (Fig. 2a and Supplementary Fig. 14) exhibit no obvious variation in the morphology after intercalation, as compared with those of the initial SnS_2_/rGO hybrid. The atomic resolution STEM-HAADF image in Fig. 2b excludes the occurrence of Pt agglomeration on the SnS_2_ nanoflakes after the solution-based intercalation. The lattice spacing is 0.324 nm and agrees well with the (100) spacing of SnS_2_ (JCPDS 00-050-0795). This unchangeable structure is confirmed by different STEM-HAADF images obtained from different areas, where no Pt bright spots are discovered as well (Supplementary Fig. 15). The energy-dispersive X-ray spectroscopy (EDS) result in Fig. 2c verifies the presence of Pt, and the elemental mapping in Supplementary Fig. 16 confirms the homogeneous distribution of Pt in the sample. In contrast, Pt nanoparticles tend to form in Pt/rGO synthesized under the same intercalation condition but with pure rGO as the support (Supplementary Fig. 17). Also, dense Pt particles are inclined to cover the as-synthesized thick SnS_2_ surface without rGO support after intercalation, rather than locating in the vdW gap (Supplementary Fig. 18). The high-resolution XPS of Pt 4 *f* confirms that the zero valence and low valence state features for Pt/rGO and thick Pt-SnS_2_, respectively. To directly validate the existence of Pt atoms in the SnS_2_ nanoislands on rGO, vertical-aligned SnS_2_ nanosheets with only a couple of nanometer lateral size were selected to observe. As depicted in the STEM-HAADF image in Fig. 2d, there are no spots noticed in the vdW gap while apparent bright spots of Pt can be observed after intercalation as shown in Fig. 2e. The comparison of XRD data in Supplementary Fig. 19 also validates the successful intercalation of Pt atoms to SnS_2_/rGO.

To compensate for the small sampling of Pt-SnS_2_/rGO in the (S)TEM analysis, XPS and XAFS analyses were conducted to acquire the macroscopic averaged structural information. The full XPS spectrum (Supplementary Fig. 20) substantiates the presence of Sn, S, C, O, and Pt in the sample. The Pt 4*f*_5/2_ peak locates at 75.3 eV and the Pt 4*f*_7/2_ peak locates at 72.0 eV, respectively, referring to a weak oxidation state of Pt (Pt^0^ ^38,39^ < Pt^δ+^<Pt^2+^ ^40^), lower than that of Pt-N-C, which is another Pt SAC with stronger bonding between Pt and the host (Supplementary Fig. 21a). The fitting of the oxidation state of Pt for Pt-SnS_2_/rGO is shown in Supplementary Fig. 21b according to the binding energy of the Pt 4*f*_5/2_ peak, exhibiting a Pt^1.2+^ oxidation state. The X-ray absorption near-edge structure (XANES) analysis for the Pt L_3_ absorption edge in Fig. 2g demonstrates that the white-line intensity of Pt-SnS_2_/rGO is higher than that of Pt foil and significantly lower than that of PtO_2_. The oxidation state of Pt in Pt-SnS_2_/rGO can also be fitted as 1.22 by integrating the white-line area (Supplementary Fig. 22), in good consonance with that of XPS fitting (Fig. 2h). Therefore, the charge transfer between intercalated Pt atoms and SnS_2_ host is relatively weak, referring to an unsaturated bonding. The extended XAFS (EXAFS) spectra in R space (Fig. 2i) reveal Pt-SnS_2_/rGO has only one prominent peak at 1.89 Å located between PtO_2_ and Pt foil, corresponding to the Pt-S bond without any Pt-Pt and Pt-Sn bonding, which clearly manifests that the single Pt atoms have been stabilized by the coordinated sulfur atoms rather than forming any Pt agglomeration or adsorbed on the top surface of SnS_2_ islands. The best-fitting analysis of the experimental curve in Supplementary Fig. 23 distinctly indicates that the single Pt atoms are each coordinated by four S atoms, consistent with the calculated structure. These results explicitly demonstrate that the intercalated Pt species are in the form of single atoms in the vdW gap of SnS_2_ via weak coordination with neighboring sulfur atoms. Moreover, the Pt atoms are stable no matter when the Pt-SnS_2_/rGO sample is heated or immersed in acid or alkali solutions (Supplementary Fig. 24). Apart from Pt-SnS_2_/rGO, Cu, Ni, Pd-SnS_2_/rGO were also synthesized via the same intercalation method. The TEM data of these contrast samples are displayed in Supplementary Fig. 25.

### Electrochemical characterization of HER catalytic activity

**Fig. 3:**
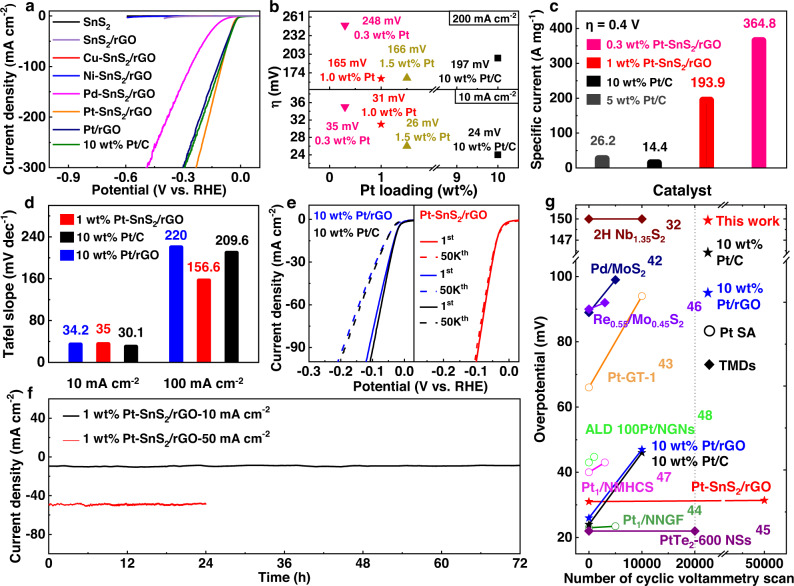
Electrochemical performance. **a** Linear sweep voltammetry (LSV) curves of 1 wt% Pt-SnS_2_/rGO, Pd-SnS_2_/rGO, Ni-SnS_2_/rGO, Cu-SnS_2_/rGO, pure SnS_2_/rGO, pure SnS_2_, 10 wt% Pt/rGO and 10 wt% Pt/C with a scan rate of 10 mV s^−1^ in 0.5 M H_2_SO_4_ solution without iR compensation. **b** Overpotentials at 10 mA cm^−2^ and 200 mA cm^−2^ of Pt-SnS_2_/rGO with three different Pt loadings (~0.3 wt%, 1.0 wt% and 1.5 wt%, respectively) and commercial 10 wt% Pt/C. **c** The Pt-mass activities of 0.3 wt% and 1 wt% Pt-SnS_2_/rGO, 5 wt% Pt/C, and 10 wt% Pt/C. **d** Tafel plots of 1 wt% Pt-SnS_2_/rGO, 10 wt% Pt/rGO, and 10 wt% Pt/C at 10 mA cm^−2^ and 100 mA cm^−2^. **e** Polarization curves of 1 wt% Pt-SnS_2_/rGO, 10 wt% Pt/rGO, and 10 wt% Pt/C before and after catalytic testing cycles. **f** Current density versus time (i–t) curve of 1 wt% Pt-SnS_2_/rGO recorded for 72 h and 24 h at a constant overpotential of 50 mV and 88 mV vs RHE, respectively. **g** Comparison of the overpotential increase after a certain number of cyclic voltammetry scans between our 1 wt% Pt-SnS_2_/rGO, 10 wt% Pt/rGO, 10 wt% Pt/C, other Pt single-atom catalysts, and 2D transition metal dichalcogenides (TMDs) reported in literatures.

The electrocatalytic activities of the SA-intercalated SnS_2_/rGO samples were investigated using HER as a probe reaction. The electrocatalytic HER performances of four kinds of metal atom-intercalated SnS_2_/rGO, pure SnS_2_/rGO, pure SnS_2_, Pt/rGO (Pt~10 wt%), and 10 wt% Pt/C (Fig. 3a and Supplementary Fig. 26a) were evaluated by the linear sweep voltammetry (LSV) curves with a constant scan rate of 10 mV s^−1^ at 25 °C without iR compensation. The almost horizontal line of pure SnS_2_ is strongly indicative of the inactive nature of pristine SnS_2_. Intercalation with Cu and Ni into SnS_2_/rGO exhibits poor HER activity similar to pristine SnS_2_/rGO. In sharp contrast, intercalation of noble metal atoms including Pt and Pd substantially reduces the overpotential. Specifically, the Pt-SnS_2_/rGO sample with ~1 wt% Pt content shows the most drastic increase in catalytic activity with an overpotential of 31 mV versus reversible hydrogen electrode (RHE) at a current density of 10 mA cm^−2^. IR compensation negligibly changes the overpotential of Pt-SnS_2_/rGO while increasing the current density at high voltage (Supplementary Fig. 27).

We further investigated the effect of intercalated Pt loading on the catalytic performance (Fig. 3b). We found that a trace amount of Pt (~0.3 wt%) could significantly reduce the overpotential to 35 mV while ~1.5 wt% Pt was capable of achieving an ultralow overpotential of 26 mV at a current density of 10 mA cm^−2^. For a high current density of 200 mA cm^−2^, the samples with ~1.0 wt% and ~1.5 wt% Pt show similar overpotential, both superior to the commercial 10 wt% Pt/C. Furthermore, the mass-specific activities based on Pt of Pt-SnS_2_/rGO (~1.0 wt%) and Pt-SnS_2_/rGO (~0.3 wt%) for HER at an overpotential of 0.4 V are 193.9 A mg^−1^ and 364.8 A mg^−1^, respectively, which are 13.5 and 25.3 times higher than that of 10 wt% Pt/C (Fig.3c and Supplementary Fig. 28), manifesting that the single-atom Pt species in the vdW gap of SnS_2_ can boost the HER catalytic activity. The Tafel plots derived from the LSV curves of 1 wt% Pt-SnS_2_/rGO, 10 wt% Pt/rGO, and 10 wt% Pt/C are shown in Fig. 3d and Supplementary Fig. 26b, which reveal slopes of 35.0 mV dec^−1^ for 1 wt% Pt-SnS_2_/rGO and 34.2 mV dec^−1^ for 10 wt% Pt/rGO, approaching that of 10 wt% Pt/C (30.1 mV dec^−1^) at 10 mA cm^−2^. Significantly, our 1 wt% Pt-SnS_2_/rGO shows the smallest Tafel slope of 156.6 mV dec^−1^ at a high current density of 100 mA cm^−2^. This low Tafel slope arises from the introduction of active Pt single atoms into the vdW gap and the formation of an interconnected conducting network via the rGO support, which enables electrons to transport rapidly from the electrode to the less-conducting SnS_2_. The turnover frequency (TOF) curves and the corresponding calculation formula of the Pt-SnS_2_/rGO catalysts with different Pt loading and the contrast 10 wt% Pt/C sample are shown in Supplementary Fig. 29, indicating the high performance of Pt-SnS_2_/rGO. Electrochemical impedance spectroscopy (EIS) was used to characterize the electron conductivity for 1 wt% Pt-SnS_2_/rGO, 10 wt% Pt/rGO, SnS_2_/rGO, and 10 wt% Pt/C (Supplementary Fig. 30). The simulated equivalent electrical circuit is also displayed in which *R*_*ct*_ is interfacial charge-transfer resistance. The semicircle diameter of Pt-SnS_2_/rGO is smaller than that of 10 wt% Pt/rGO and SnS_2_/rGO, clarifying the low resistance of rapid interfacial charge transfer and fast HER kinetics of Pt-SnS_2_/rGO. We expect that intercalating the Pt atoms into the vdW gap between SnS_2_ layers could stabilize and protect the Pt atoms from direct protruding into electrolytes while H^+^ still can migrate in the vdW gap and easily be adsorbed to the Pt atoms, and thus help to improve the durability of the catalyst during electrocatalysis (Supplementary Fig. 31). Cycling performance and long-time durability testing were carried out to validate this conjecture. The LSV curves of 1 wt% Pt-SnS_2_/rGO, 10 wt% Pt/rGO, and 10 wt% Pt/C were measured before and after catalytic cycling (Fig. 3e and Supplementary Fig. 32). The 1 wt% Pt-SnS_2_/rGO sample exhibited no evident change even after 50,000 cycles, whereas the 10 wt% Pt/rGO and 10 wt% Pt/C showed an obvious loss in activity after just 10,000 cycles due to the dissolution of Pt into the reaction solution^41^. This result suggests that the intercalated catalyst is much more stable than the reference Pt nanoparticle catalysts. The steady chronopotentiometry curve of the 1 wt% Pt-SnS_2_/rGO tested at a constant overpotential of 50 mV displayed no conspicuous decay of the current after 72 h (Fig. 3f), further affirming the ultrahigh stability. Even at a high current density of 50 mA cm^−2^, this catalyst exhibited excellent cycle stability within 24 h. In addition, the overpotential of our 1 wt% Pt-SnS_2_/rGO catalyst only increased by 0.1 μV per cycle at a current density of 100 mA cm^−2^, which is significantly smaller than that of any other catalyst systems containing Pt single atoms on various carbon-based materials or 2D transition metal dichalcogenides (TMDs) reported (Fig. 3g)^32,42–48^. The above virtues of the 1 wt% Pt-SnS_2_/rGO, including CV and i-t long-term stability, are superior to most Pt-based HER catalysts in previous literature (Fig. 3g, Supplementary Fig. 33, and Tables 2 and 3).

Ex-situ structural characterization was further carried out to analyze the morphology and the valence state of the cycled sample of 1 wt% Pt-SnS_2_/rGO. The TEM images (Supplementary Fig. 34) suggest that the average diameter, crystal structure, and distribution of the Pt-SnS_2_ nanoflakes on rGO were well preserved after the electrocatalytic testing. The XPS spectrum of the sample after 50,000 cycles exhibits two broad peaks at 75.7 and 72.5 eV, respectively, corresponding to Pt 4*f*_5/2_ and Pt 4*f*_7/2_ (Supplementary Fig. 35), which are similar to the spectroscopic features from the as-synthesized sample. These results further confirm the remarkable structural stability of the 1 wt% Pt-SnS_2_/rGO catalyst.

To manifest the university of high catalytic performance, HER data of this Pt-SnS_2_/rGO catalyst in alkaline and neutral conditions are shown in Supplementary Fig. 36. The overpotentials are 59 mV and 54 mV, respectively, which are also relatively desirable performances. After 10,000 cycles, the LSV curves both maintain good performance, and especially, the stability under neutral condition is admirable.

### Theoretical calculations

**Fig. 4:**
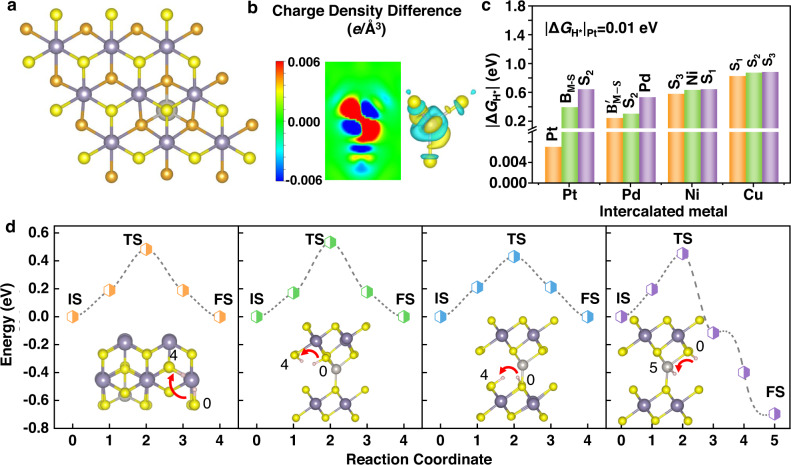
The theoretical calculation of SA-intercalated SnS_2_. **a** The relaxed Pt-intercalated SnS_2_ crystal structure. Yellow: S atoms on the upper surface of SnS_2_. Orange: S atoms on the lower surface of SnS_2_. Purple: Sn atoms of SnS_2_. Gray: the intercalated Pt atom. **b** The localized charge density difference distribution of Pt-SnS_2_. Isovalue is 0.006 *e*/Å^3^. **c** The smallest three Gibbs free energy (|Δ*G*_H*_|) values of Pt, Pd, Ni, and Cu-SnS_2_, respectively. **d** The energy diagrams of different H-migration pathways on Pt-intercalated SnS_2_. IS initial state, TS transitional state, FS final state. The insets illustrate the migration of H from IS to FS.

DFT calculations were carried out to figure out the interaction between Pt and SnS_2_, as well as the mechanism of hydrogen evolution on Pt-intercalated SnS_2_. As depicted in Fig. 4a and the insets in Fig. 4d, the intercalated SnS_2_ crystal remains the hexagonal atomic configuration with AA stacking, and each of the metal atoms in the vdW gap is coordinated with four sulfur atoms. The strong localization of charge different distribution with small amplitude shown in Fig. 4b indicates that weak bonding between intercalated Pt and SnS_2_, which is corroborated by the band structure change in Supplementary Fig. 1 showing localized defect states contributed by Pt atoms mainly emerge inside the band gap of SnS_2_. Meanwhile, the Bader charge analysis shows that in Pt-intercalated SnS_2_ the transferred charge from Pt to SnS_2_ is only 0.03 *e*, consistent with the characteristics of weak bonding.

To understand the chemical deposition of Pt atoms on rGO and SnS_2_, we calculated the formation energies of Pt atoms on these two substrates against the cluster formation taking Pt_13_ as a reference for simplicity (Supplementary Fig. 37). It can be seen that the formation energies for Pt adsorption on rGO (Supplementary Fig. 38 and Table 4) are all larger than zero, suggesting the agglomeration of Pt atoms to form clusters is preferred. In contrast, the formation energy of Pt-intercalated SnS_2_ is −1.53 eV, indicating the intercalation is more favorable than the adsorption on the rGO surface, which is consistent with the experimental results above. Additionally, the formation energy of Pt-intercalated SnS_2_ is lower than those of Pt adsorption on the SnS_2_ surface (Supplementary Fig. 39), excluding the presence of Pt atoms on surface.

We then calculated the Gibbs free energy of hydrogen adsorption (|Δ*G*_H*_|) and energy barriers of hydrogen migration on the intercalated catalyst. Seven possible active sites in SA-intercalated SnS_2_ were evaluated, including M, S_1_, S_2_, S_3_, B_M-S_, B’_M-S_, and B_S-S_, respectively (Supplementary Fig. 40). Comparing the most desirable active sites in each SA-intercalated SnS_2_ structure (Fig. 4c and Supplementary Fig. 41), the Pt site in Pt-SnS_2_ explicitly presents the lowest value of|Δ*G*_H*_|(0.01 eV), which should lead to superior HER catalytic activity. B’_M-S_ (|Δ*G*_H*_|is 0.25 eV), S_3_ (|Δ*G*_H*_|is 0.59 eV), and S_1_ (|Δ*G*_H*_|is 0.83 eV) sites are the most active sites for Pd, Ni, and Cu-SnS_2_, respectively, in accord with the catalytic activities of these samples shown by the LSV curves. These theoretical results also suggest that the intercalators could also activate the catalytic activity of the typically inert SnS_2_ crystal. Energy diagrams of four different H-migration pathways on Pt-SnS_2_ are illustrated in Fig. 4d, including S-S pathway on the outside surface, S-S pathways inside the vdW gap, and S-Pt pathway. The transitional states of different H-migration pathways are shown in Supplementary Fig. 42. The energy barriers for all four pathways are between 0.43 and 0.53 eV. Such low energy barriers make it easy to transfer protons on the surface and interlayers of Pt-SnS_2_, providing abundant H atoms to adsorb onto the active Pt atoms, which remarkably improves the HER performance of this intercalated catalyst.

## Discussion

In summary, we designed a class of SACs with different interaction types by confining metal single atoms into the vdW gap of a 2D material, especially high-activity noble metal SACs. The relatively weak bonding between noble metal atoms and the 2D material could endow the metal single atoms catalytic properties closer to the intrinsic activity of their metallic bulk counterparts compared to the ones with strong chemical bonding. More importantly, the intercalated metal single atoms within the vdW gap can be well protected by the layered host, conferring unusually high stability compared to the physically adsorbed SACs on the surface. As a demonstration, we show that the Pt-SnS_2_/rGO nanocomposite with 1 wt% Pt loading shows superior catalytic performance in HER at a high current density to that of commercial Pt/C catalyst with 10 times higher Pt loading. Our study opens an avenue to design and explore a class of single-atom-like catalysts through the intercalation of various metal atoms into a wide range of 2D materials for practical applications including and beyond electrocatalysis.

## Methods

### Synthesis of atomically thin SnS_2_

SnS_2_ was grown via a CVD method by using sulfur powder (S, 99.5%, Sigma-Aldrich), tin dioxide (SnO_2_, 99.99%, Alfa), and potassium iodide (KI, 99.998%, Alfa) as precursors. Specifically, 10 mg SnO_2_ and 1 mg KI powder were placed at the center of the furnace with a SiO_2_/Si substrate on the top. 200 mg S was placed in an alumina boat upstream. The temperature of the furnace firstly ramped up to 600 °C for 5 min at a heating rate of 40 °C min^−1^ for the growth. During the growth, the temperature of S was about 200 °C. The entire sintering process was completed under the condition of Ar atmosphere (99,999%) with a flow of 50 sccm. SnS_2_ with various thicknesses could be obtained by controlling the growth time.

### Synthesis of SnS_2_/rGO and rGO

The SnS_2_/rGO hybrid nanostructure was achieved by a one-pot hydrothermal reaction using stannic chloride pentahydrate (SnCl_4_·5H_2_O, 98%, Sigma-Aldrich), thiourea (CH_4_N_2_S, 99%, Acros) and graphene oxide (GO, Nanjing Xianfeng Nano) as precursors. Specifically, 20 mg GO was added to 20 mL of deionized water (DI) and sonicated for 2 h. Then, 130 mg SnCl_4_·5H_2_O and 320 mg CH_4_N_2_S were poured into the solution and stirred for 1 h. Finally, the solution was transferred to a 50 mL Teflon-lined stainless-steel autoclave and heated at 190 °C for 16 h. When the autoclave cooled down to room temperature, the as-synthesized SnS_2_/rGO hybrid nanosheets were washed with DI water and ethanol three times. The SnS_2_/rGO nanosheets were finally freeze-dried for 24 h. As a contrast sample, pure rGO nanosheets were acquired in the same way in the absence of SnCl_4_·5H_2_O and CH_4_N_2_S.

### Intercalation of Pt, Pd, Ni, and Cu to CVD-grown SnS_2_

A solvent-based method was used to intercalate Pt, Pd, Ni, and Cu atoms into the vdW layers of SnS_2_. Acetone (99%, Acros) was used as the solvent and ammonium hexachloroplatinate (IV, 43.4% Pt, Alfa) and glucose (99%, Alfa), ammonium tetrachloropalladate (II, 29% Pd, Alfa) and ascorbic acid (99%, Acros), nickel(II) chloride (99.99%, Acros) and sodium borohydride (98%, Acros), tetrakis(acetonitrile) copper(I) hexafluorophosphate (98%, Tci) reacted as the precursors to provide intercalated Pt, Pd, Ni, and Cu atoms, respectively. Typically, CVD-grown SnS_2_ on Si/SiO_2_ was put into a vial with 20 mL acetone and 3~5 mg corresponding precursors mentioned above. A relatively slow rate for dispersing the metal atoms in the solution is the key to successful intercalation, indicating the precursor concentration and reaction temperature should be appropriate. Moreover, the density of metal atoms inserted in the SnS_2_ vdW layers can be adjusted by changing the concentration of precursors. The intercalation processes were conducted at a temperature between 50–120 °C for 1–3 h in Ar gas (99.99%). After the reaction, the samples were further rinsed with hot acetone and dried in the air.

### Synthesis of SA-SnS_2_/rGO and Pt/rGO

The SA-intercalated SnS_2_/rGO (SA: Cu, Ni, Pd, Pt) can be synthesized according to the above intercalation method. Typically, 40 mg SnS_2_/rGO was firstly dispersed in a 15 mL acetone solution in a 20 mL vial. Then, 3 mg ammonium hexachloroplatinate (IV) and 5 mg glucose, 3 mg ammonium tetrachloropalladate (II) and 5 mg ascorbic acid, 3 mg nickel(II) chloride and 5 mg sodium borohydride, 3 mg tetrakis(acetonitrile) copper(I) hexafluorophosphate were added to each vial and stirred at 50–120 °C for 1–3 h in Ar gas, respectively. After the intercalation reaction, the samples were filtered, rinsed by hot acetone and then freeze-dried, marked as Pt-SnS_2_/rGO, Pd-SnS_2_/rGO, Ni-SnS_2_/rGO, and Cu-SnS_2_/rGO, respectively. The contrast sample Pt/rGO was synthesized in the same process using rGO instead of SnS_2_/rGO.

### Material characterizations

X-ray powder diffraction (XRD) patterns were obtained on an X-ray diffractometer (Rigaku D/max2500PC) equipped with Cu Kα radiation for the 2-Theta range of 10°−80°. Field emission scanning electron microscope (FE-SEM, Hitachi SU8020) observations were used to characterize the morphology with energy-dispersive spectrometry (EDS). Transmission electron microscope (TEM) observations were examined under the JEOL JEM-2100F. High-resolution scanning transmission electron microscopy high-angle annular dark-field (STEM-HAADF) imaging of the cross-sectional sample was conducted on the Nion HERMES-100 with a C3/C5 corrector, operated at 100 kV. The collection angle of the HAADF detector was 92–210 mrad and the probe forming angle is ~30 mrad. Raman spectroscopies (Renishaw inVia) of as-prepared samples were performed at Horiba Jobin-Yvon LabRAM Aramis Raman microscopy. XPS experiments were carried out on an ESCALAB 250Xi instrument.

### Electrochemical measurements

The electrocatalytic HER activities were measured in an Ar-saturated H_2_SO_4_ solution (0.5 M) using a standard three-electrode configuration on CHI 760E electrochemical workstation. The working electrode was a glassy carbon electrode (3 mm in diameter) coated with the catalysts. Graphite rod and Ag/AgCl (saturated KCl solution) were used as the counter electrode and the reference electrode, respectively. All the measured potentials were referred to reverse hydrogen electrode (RHE) with the equation of E(RHE) = E(Ag^+^/AgCl) + 0.204 V + 0.0591 pH. All catalyst (Pt-SnS_2_/rGO, Pd-SnS_2_/rGO, Ni-SnS_2_/rGO, Cu-SnS_2_/rGO, pure SnS_2_, 10 wt% Pt/rGO, 10 wt% Pt/C) inks were prepared by homogeneously dispersing corresponding 4 mg catalysts and 5 μL of 5 wt% Nafion solution in 1 mL water/ethanol (4:1 v/v) solution. The above 5 μL slurry was coated on glassy carbon electrode (3 mm in diameter) and dried at 25 °C for 12 h as the working electrode. The catalyst loading on a glassy carbon electrode (disk geometric area, 7.076 mm^2^) was calculated as approximately 0.281 mg cm^−2^. The Pt loading of 1 wt% Pt-SnS_2_/rGO, 10 wt% Pt/rGO, and 10 wt% Pt/C was about 2.81, 28.1, and 28.1 μg cm^−2^, respectively. All measured potentials in all tests were no iR compensated unless otherwise specified. Linear sweep voltammetry (LSV) curves and Tafel curves were recorded at the scan rate of 10 mV s^−1^ from 0.104 to −0.596 V (vs RHE) at 25 °C in the incubator. The working electrode was pre-activated by repeating the potential scan from 0.104 to −0.596 V (vs RHE) at a scan rate of 10 mV s^−1^ for 200 CV cycles at 25 °C in the incubator. The durability tests were performed by repeating the potential scan from 0.104 to −0.296 (vs RHE) at a scan rate of 50 mV s^−1^ for 10,000 or 50,000 cycles of cyclic voltammetry (CV) at 25 °C in the incubator. Chronoamperometric characterization was performed in an Ar-saturated 0.5 M H_2_SO_4_ solution at an overpotential 50 mV (vs RHE) under 10 mA cm^−2^ for 72 h and 88 mV (vs RHE) under 50 mA cm^−2^ for 24 h, respectively. Electrochemical impedance spectroscopy (EIS) measurements were conducted in the frequency range of 0.01–100,000 Hz at the potential of −0.200 V (vs. RHE) with a perturbation of 5 mV.

### Computational methods and details

All first-principles calculations were performed using Vienna ab initio simulation package (VASP)^1^ with the Tkatchenko-Scheffler mothed^2^ describing vdW interaction. Structural models were constructed by a 2 × 2 cell of SnS_2_ which was intercalated with one metal atom between layers. The vacuum layer thickness of 15 Å was chosen to avoid interlayer interaction. The structural optimization was performed with convergence criteria for energy and force set as 10^−5^ eV/Å and 0.01 eV/Å, respectively. The cutoff energy of the plane-wave basis was set to 500 eV, and the Monkhorst-Pack 9 × 9 × 1 mesh was selected to sample the reciprocal Brillouin zone. The formation energy of Pt SA is defined as E_f_ = E_tot_-E_ref_-μ_Pt_^3,4^, where E_tot_ and E_ref_ are the total energies of SnS_2_ systems with and without Pt adsorption, respectively. The μ_Pt_ is the chemical potential of Pt in the magic-number Pt_13_ cluster for simplicity. The free energy (G) was obtained as G = E_0_ + E_ZPE_-TS, with ground-state energy (E_0_) and zero-point vibrational energy (E_ZPE_) from DFT calculations. The entropy (S) for adsorbed H was calculated following well-established procedure^5^, whereas the thermodynamics for H_2_ molecules were taken from standard tables.

## Supplementary information

Supplementary Information

## Data Availability

The data supporting this study are available within the paper and the Supplementary Information. Source data are provided with this paper.

## Source data

Source Data
