# Supplementary material for: Single atom catalysts in Van der Waals gaps
Source: Nat Commun. 2022 Nov 11;13:6863. doi: 10.1038/s41467-022-34572-3 (PMC9652421; doi:10.1038/s41467-022-34572-3)
Supplement: Supplementary file 1 — Supplementary Information [file 41467_2022_34572_MOESM1_ESM.pdf]

Supplementary Materials for

Single atom catalysts in Van der Waals gaps

Huaning Jiang[1], Weiwei Yang[1, 2], Mingquan Xu[3], Erqing Wang[4], Yi Wei[5], Wei Liu[1], Xiaokang Gu[1], Lixuan Liu[1], Qian Chen[1], Pengbo Zhai[1, 6], Xiaolong Zou[4], Pulickel M. Ajayan[7], Wu Zhou[3*], Yongji Gong[1, 8*]

[1]School of Materials Science and Engineering, Beihang University, Beijing 100191, China.

[2]School of Materials and Chemistry, University of Shanghai for Science and Technology, 200093, Shanghai, P. R. China.

[3]School of Physical Sciences and CAS Key Laboratory of Vacuum Physics, University of Chinese Academy of Sciences, Beijing 100049, China.

[4]Shenzhen Geim Graphene Center and Low-Dimensional Materials and Devices Laboratory, Tsinghua-Berkeley Shenzhen Institute, Tsinghua University, Shenzhen 518055, China.

[5]State Key Laboratory of Organic-Inorganic Composites, Beijing Key Laboratory of Electrochemical Process and Technology for Materials, Beijing University of Chemical Technology, Beijing 100029, China.

[6]College of Physics, Qingdao University, Qingdao 266071, China.

[7]School of Material Science & NanoEngineering, Rice University, Houston, Tx 77005, USA.

[8]Center for Micro-Nano Innovation of Beihang University, Beijing 100191, China.

These authors contributed equally: Huaning Jiang, Weiwei Yang, Mingquan Xu, Erqing Wang.

[*]e-mail: yongjigong@buaa.edu.cn (Y.G.) ; wuzhou@ucas.ac.cn (W.Z.).

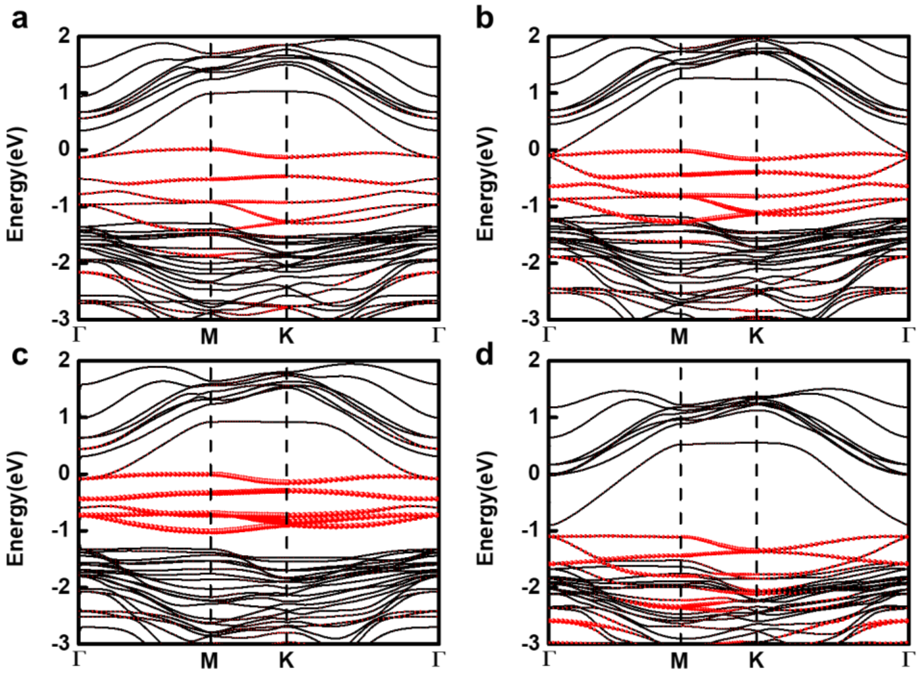

Supplementary Fig. 1 The projected band structures of SA-intercalated SnS_2. a-d, The red spheres represent the contribution of SA in Pt, Pd, Ni, and Cu-intercalated SnS_2, respectively. The Fermi level is set to zero.

It can be clearly seen that the intercalation of SA introduces defect states inside the band gap of SnS_2, and the transition of electrons from these states to conduction states could introduce new adsorption peaks, causing color change in optical images. For Pt, Pd, and Ni-intercalated SnS_2, the defect states of intercalated metal atoms mainly locate inside the band gap and are well isolated from the SnS_2 bulk states. These behaviors indicate that metal atoms are weakly bonded to the SnS_2. In contrast, for Cu-intercalated SnS_2 case the Cu orbitals hybrid with the dispersive valence bands of SnS_2, exhibiting strong coupling between Cu and SnS_2. Meanwhile, the Bader charge analysis shows that in Pt-intercalated SnS_2 the transferred charge from Pt to SnS_2 is only 0.03 e, consistent with the characteristics of weak bonding.

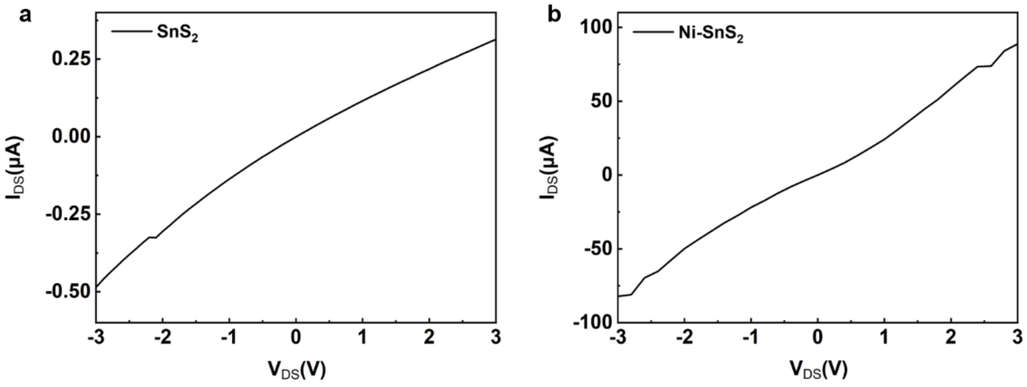

Supplementary Fig. 2 The device data. I_{DS}-V_{DS} curves at room temperature of pristine SnS_2 and Ni-SnS_2 grown by CVD. **a,** Pristine SnS_2. **b,** Ni-SnS_2.

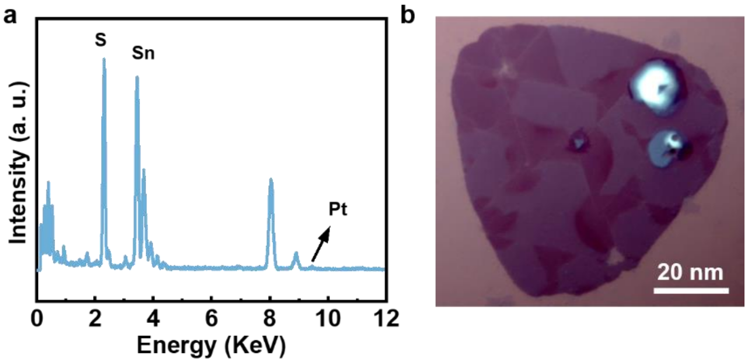

Supplementary Fig. 3 Different intercalation distribution. a, The EDS data of homogeneously intercalated SnS_2. **b,** The optical image of Pt-SnS_2 with inhomogeneous color under the same thickness.

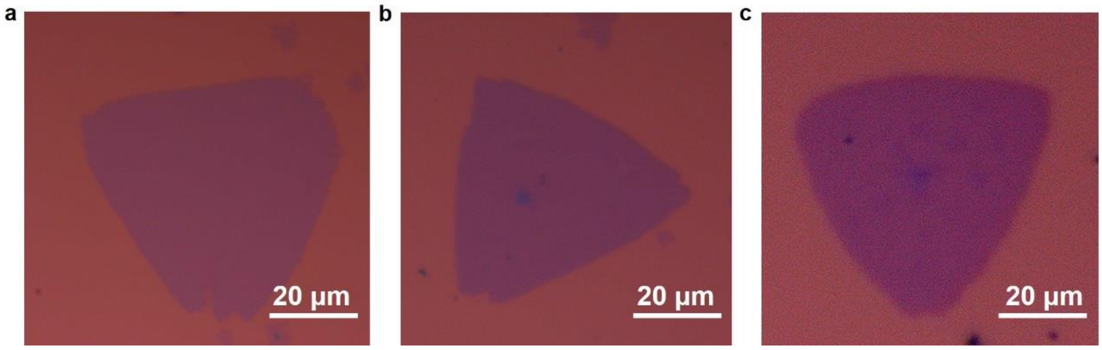

Supplementary Fig. 4 Optical images of CVD-grown SnS_2 before and after intercalation with different Pt concentrations. a, The optical image of pristine SnS_2 in size of tens of micrometers and the color is light pink. **b-c**, Optical images of Pt-SnS_2 in variable colors including deep pink (**b**) and purple (**c**) thanks to the low and high intercalated Pt loading. The different loadings are attributed to the variable concentration under the same conditions of temperature and time, that is to say, the low (0.34 mmol L^{-1}) and high (0.51 mmol L^{-1}) concentration of ammonium hexachloroplatinate, respectively. These images indicate that controlling the solution concentration can provide intercalated SnS_2 nanosheets with different loading of the intercalated guest atoms.

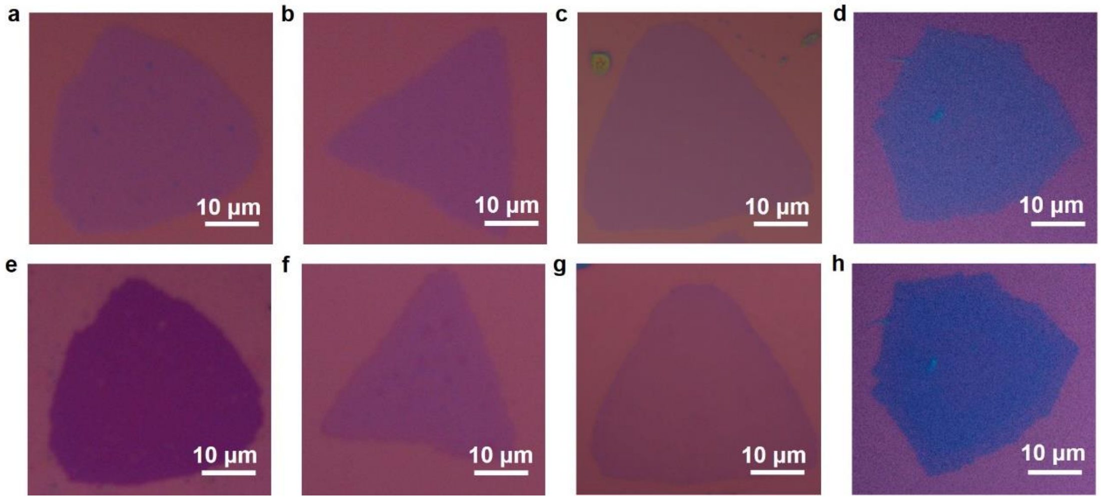

Supplementary Fig. 5 Optical images of CVD-grown SnS_2 before and after intercalation of different metal atoms. a-d, Optical images of pristine SnS_2 before intercalation. **e-h,** Optical images of the corresponding SnS_2 after intercalation by Pt, Pd, Ni, and Cu atoms, respectively. The unchanged morphology of the nanosheets verifies that intercalation does not cause structural degradation, no matter what the intercalated element is.

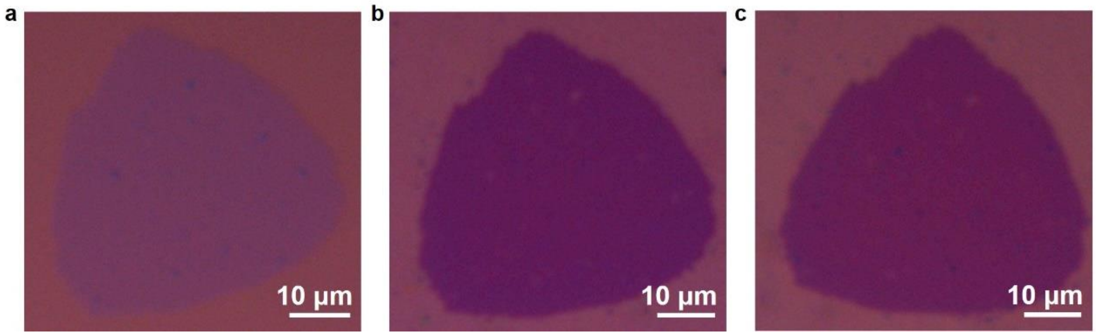

Supplementary Fig. 6 Stability test. a, The optical image of the pristine SnS_2. **b,** The optical image of Pt-SnS_2. **c,** The optical image of Pt-SnS_2 in **b** when exposed to air for 2 months. The stability of intercalated SnS_2 can be demonstrated by its unchanged color, morphology, and continuity, beneficial for practical applications.

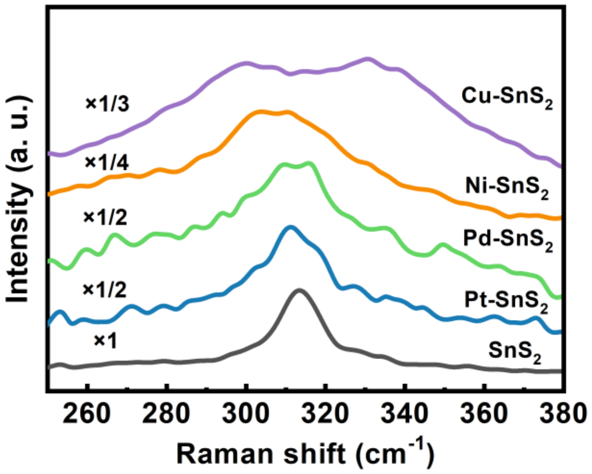

Supplementary Fig. 7 Raman spectra of pristine and intercalated CVD-grown SnS_2. The different Raman spectra indicate the successful intercalation of Pt, Pd, Ni, and Cu atoms to SnS_2 as the phonon modes of SnS_2 are affected by the intercalated metal atoms. Different from the only one Raman peak at 314 cm^{-1} of pristine SnS_2, split peaks occur in the Raman spectra of these four intercalated SnS_2 samples. In addition, Pt-intercalated SnS_2 manifests the least apparent change of Raman modes due to the weakest charge transfer, in good agreement with the DFT calculations.

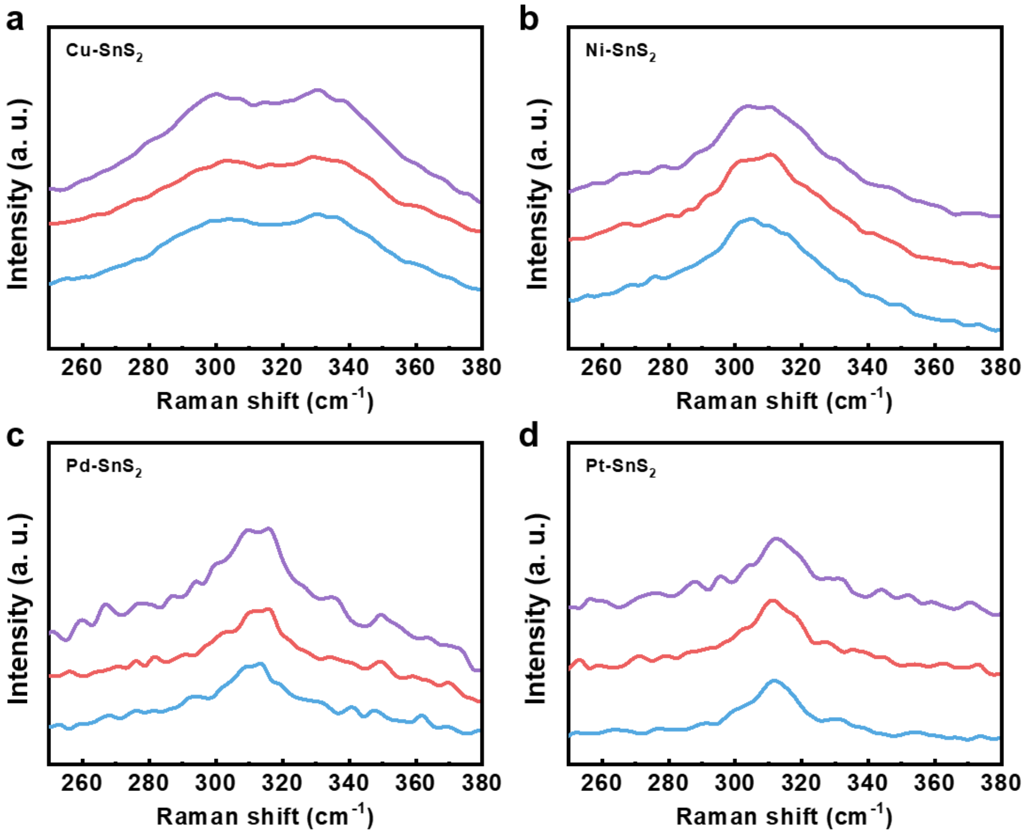

Supplementary Fig. 8 The reproducibility of Raman spectra. Raman spectra of Cu, Ni, Pd, and Pt-SnS_2 from three different spots with the same thicknesses, respectively. **a,** Cu-SnS_2. **b,** Ni-SnS_2. **c,** Pd-SnS_2. **d,** Pt-SnS_2.

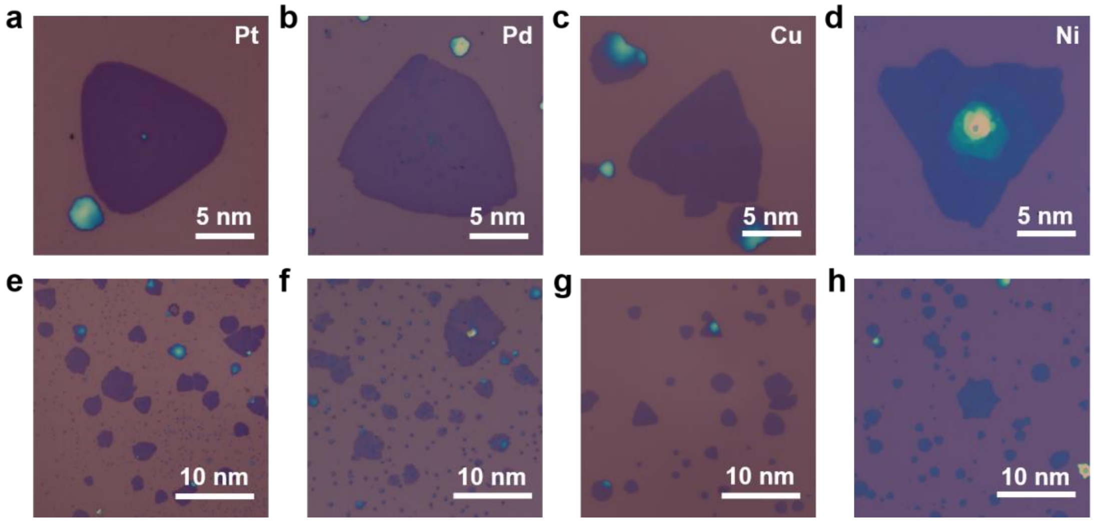

Supplementary Fig. 9 The homogeneous distribution of intercalation. a-d, The optical images of individual nanosheets of Pt, Pd, Cu, and Ni-SnS_2 with homogeneous colors, respectively. **e-h,** The larger-scale optical images of Pt, Pd, Cu, and Ni-SnS_2 samples under the same thicknesses with homogeneous colors, respectively.

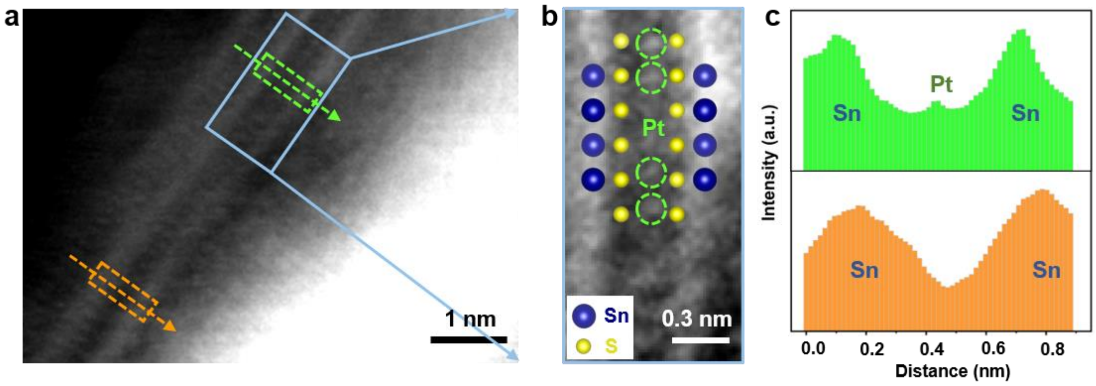

Supplementary Fig. 10 Cross-sectional STEM-HAADF images of Pt intercalated CVD-grown SnS_2. a, Cross-sectional STEM-HAADF image of Pt-SnS_2. **b,** Enlarged image of the region highlighted by the blue rectangle in **a**. **c,** Line intensity profiles obtained from the areas highlighted by the green and orange rectangles in **b**.

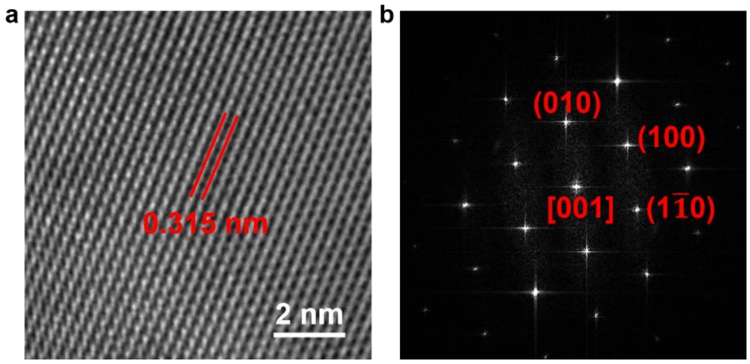

Supplementary Fig. 11 HRTEM image and the corresponding FFT pattern of Pt-intercalated CVD-grown SnS_2. a, HRTEM image of Pt-SnS_2 shows stable SnS_2 structure after intercalation with the lattice spacing coincident with pristine SnS_2. **b,** The corresponding FFT image of (a) exhibits one set of crystal lattice of SnS_2 phase in the Pt-intercalated SnS_2 sample. These results distinctly indicate that Pt atoms are uniformly distributed in the vdW gap of SnS_2, without Pt nanoparticles on the surface.

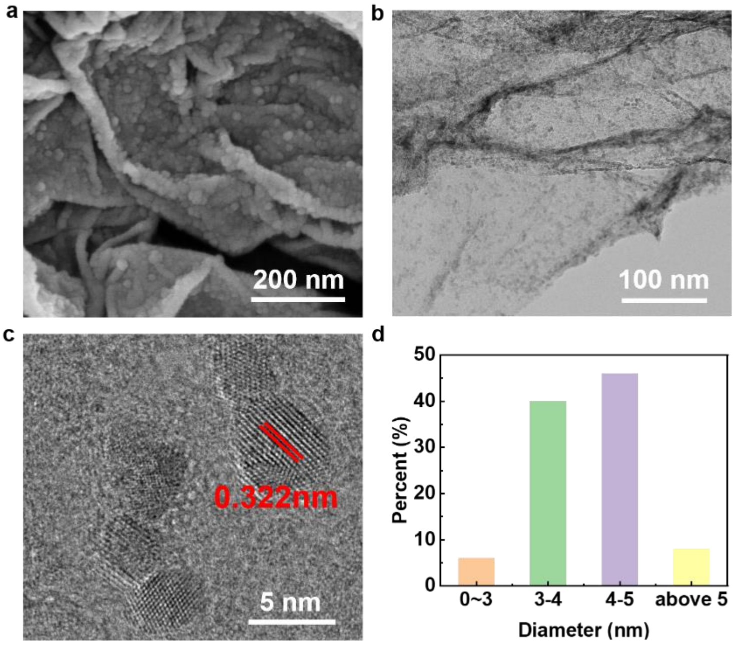

Supplementary Fig. 12 Morphological Characterizations of SnS_2/rGO. a, The SEM image of SnS_2/rGO. **b,** The TEM image of SnS_2/rGO. These images exhibit that the downsized SnS_2 nanosheets are uniformly distributed on rGO. **c,** The HRTEM image of SnS_2/rGO displays the relatively homogeneous size distribution and the hexagonal crystal structure of the SnS_2 nanosheets on rGO. **d,** The size distribution of SnS_2 on rGO manifests that the diameters of SnS_2 nanosheets are mainly 3~5 nm.

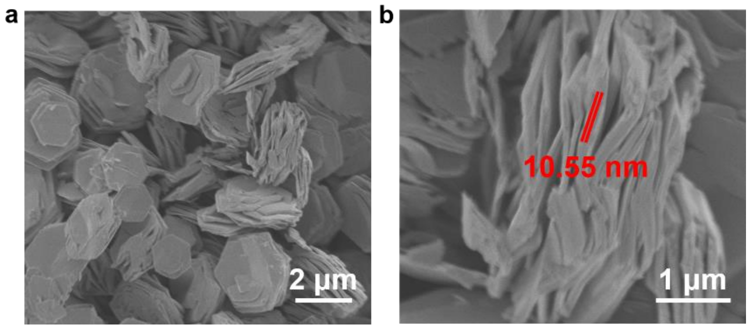

Supplementary Fig. 13 SEM images of pure SnS_2 nanoplates. a, The SEM image of stacked pure SnS_2 nanoplates synthesized without rGO. The diameters of the nanoplates are about 1.5~4 μm. **b,** The SEM image exhibits the thickness of one SnS_2 nanoplate. Under the same condition, pure SnS_2 prefers to grow into nanoplates thicker than 10 nm with a lateral size in micrometer.

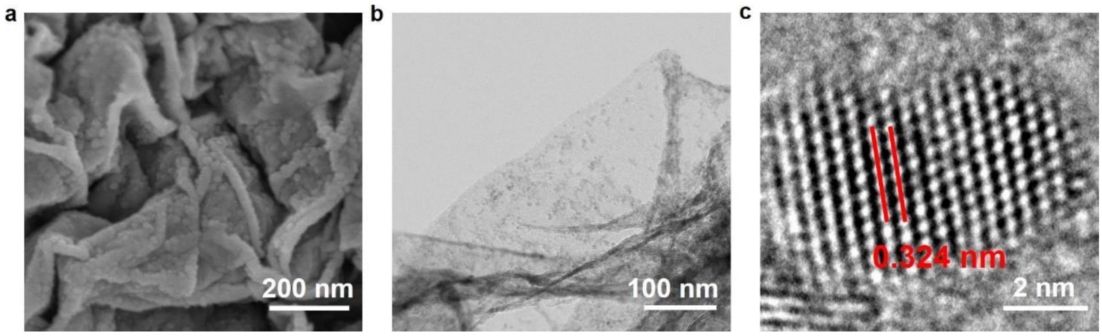

Supplementary Fig. 14 Morphological Characterization of Pt-SnS_2/rGO. a, The SEM image of Pt-SnS_2/rGO. **b,** The TEM image of Pt-SnS_2/rGO. There are no obvious variations in the distribution and morphology of the intercalated SnS_2 nanosheets on rGO compared to pristine SnS_2/rGO. **c,** The HRTEM image of Pt-SnS_2/rGO. The crystal structure is consistent with that of SnS_2, revealing that the Pt atoms are intercalated to the vdW gap, rather than doped into the crystal structure of SnS_2.

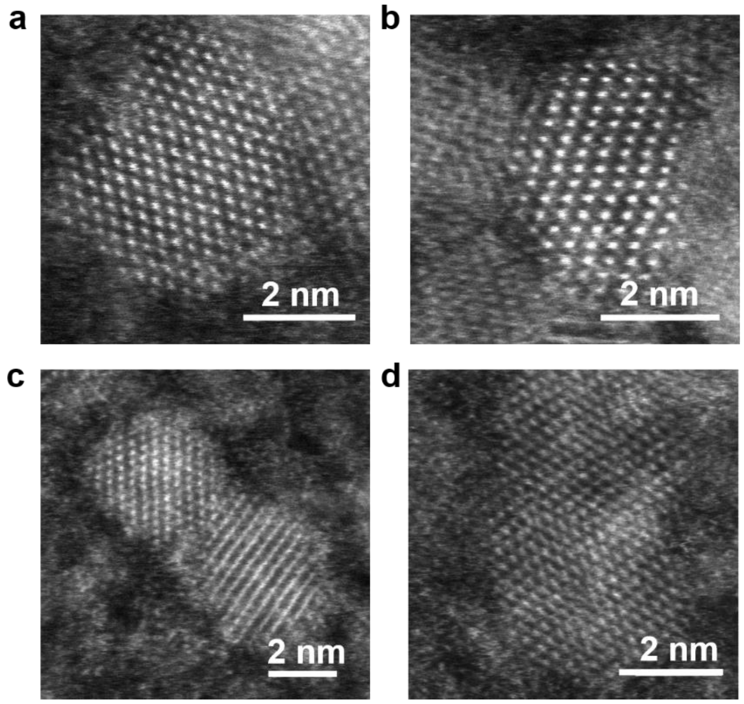

Supplementary Fig. 15 HAADF-STEM images of Pt-SnS_2/rGO. a-d, HAADF-STEM images of Pt-SnS_2 islands on rGO obtained from four different areas.

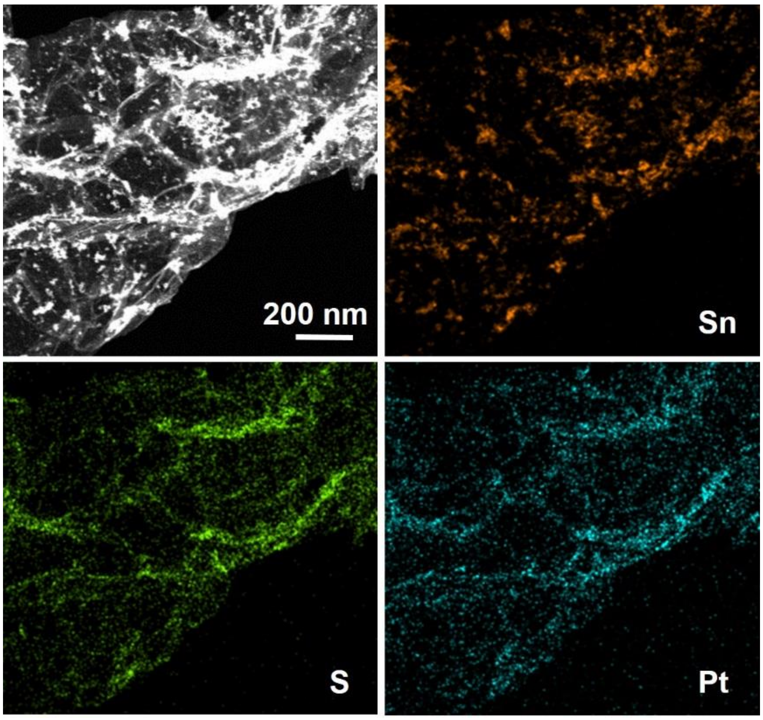

Supplementary Fig. 16 The Element mapping of Pt-SnS_2/rGO. The HAADF image of Pt-SnS_2/rGO and the corresponding element maps including Sn, S and Pt substantiate the homogeneity of Pt intercalation as the distribution of Pt is entirely in accord with SnS_2.

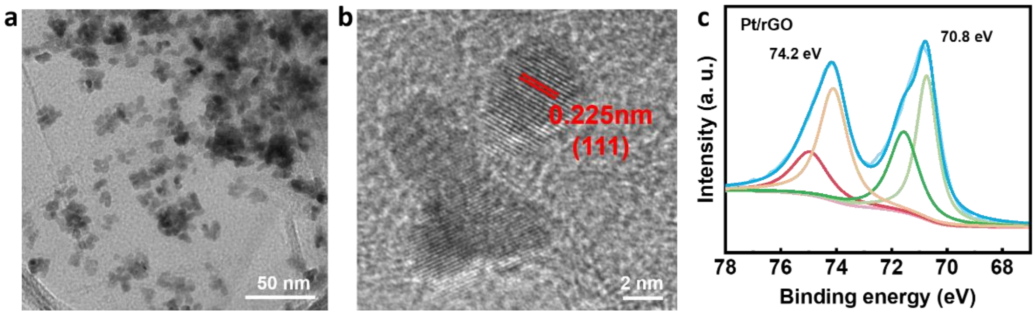

Supplementary Fig. 17 The TEM images of Pt/rGO. a, The TEM image of Pt/rGO clearly shows that dense Pt nanoparticles tend to form with nonuniform distribution when the support is pure rGO. **b,** The HRTEM image of Pt/rGO clearly exhibits the lattice spacing of Pt (111), indicating the formation of Pt nanoparticles on rGO. **c,** The high-resolution XPS of Pt 4*f* for Pt/rGO.

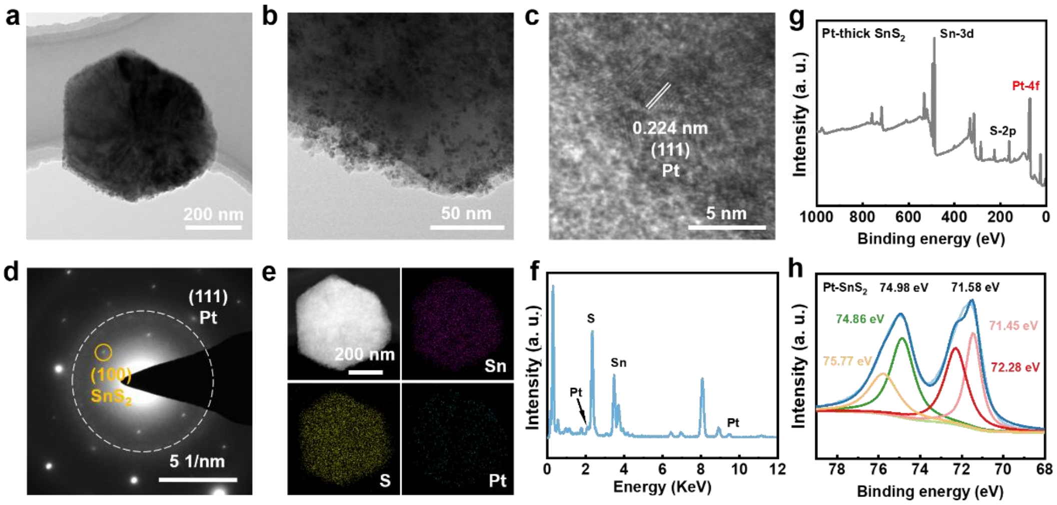

Supplementary Fig. 18 The TEM images and XPS spectra of Pt/rGO. a, The TEM image of a thick SnS_2 with Pt atoms on the surface. **b,** The magnified TEM image of the thick Pt-SnS_2. **c,** the HRTEM image of the thick Pt-SnS_2 with obvious Pt crystal structure on the surface. **d,** The SAED image of the thick Pt-SnS_2 with a set of diffraction spots of SnS_2 as well as a diffraction ring of Pt particles. **e,** The mapping of the thick Pt-SnS_2. **f,** The EDS spectrum of the thick Pt-SnS_2. **g,** The XPS spectrum of thick Pt-SnS_2. **h,** The high-resolution XPS spectrum of Pt 4*f* for thick Pt-SnS_2.The intensities of Pt peaks in (f) and (g) are different because that the shallow depth of surface detection of XPS which is only within several nms while that of EDS is up to several μms. Therefore, although the Pt content is small according to the EDS spectrum, the Pt peak in XPS is obvious as a result of the Pt particles coated on the surface.

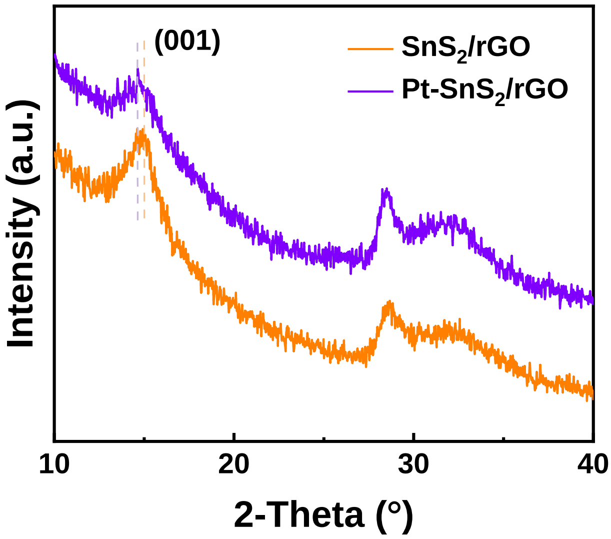

Supplementary Fig. 19 XRD results of SnS_2/rGO and Pt-SnS_2/rGO. The position shift of (001) peak indicates the intercalation of Pt atoms to SnS_2.

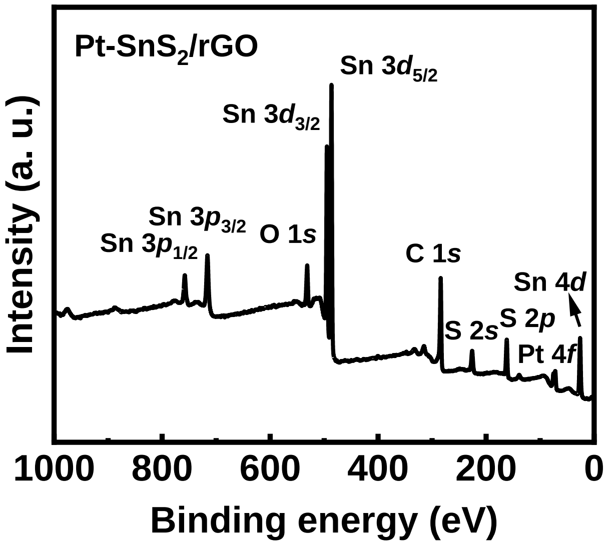

Supplementary Fig. 20 The full XPS spectrum of Pt-SnS_2/rGO. The full XPS spectrum confirms the presence of Sn, S, C, O, and Pt elements.

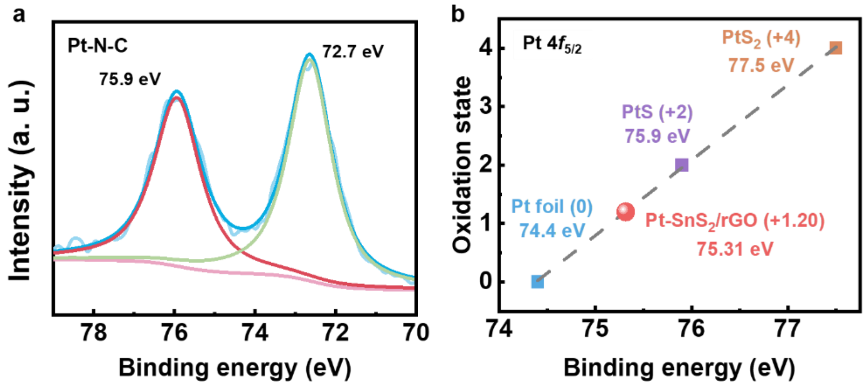

Supplementary Fig. 21 XPS data. a, The high-resolution XPS spectrum of Pt 4*f* for Pt-N-C catalyst. **b,** The fitting of the oxidation state of Pt for Pt-SnS_2/rGO from the binding energy.

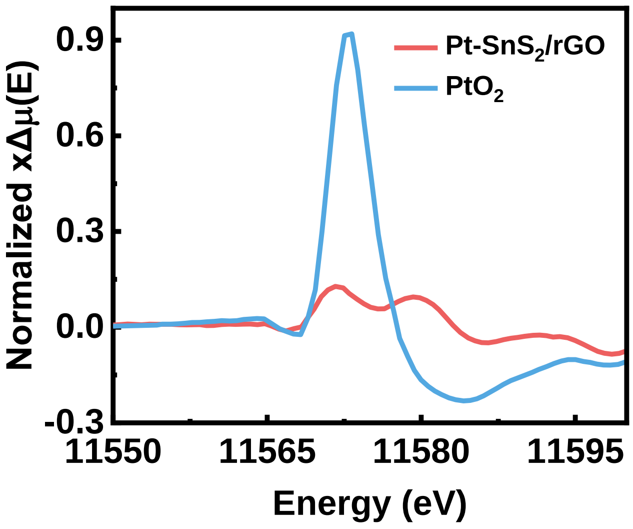

Supplementary Fig. 22 Normalized ΔXANES spectra for Pt L_3-edge using Pt foil as the reference. The oxidation states are fitted by integrating the white-line area from 11568 to 11585 eV.

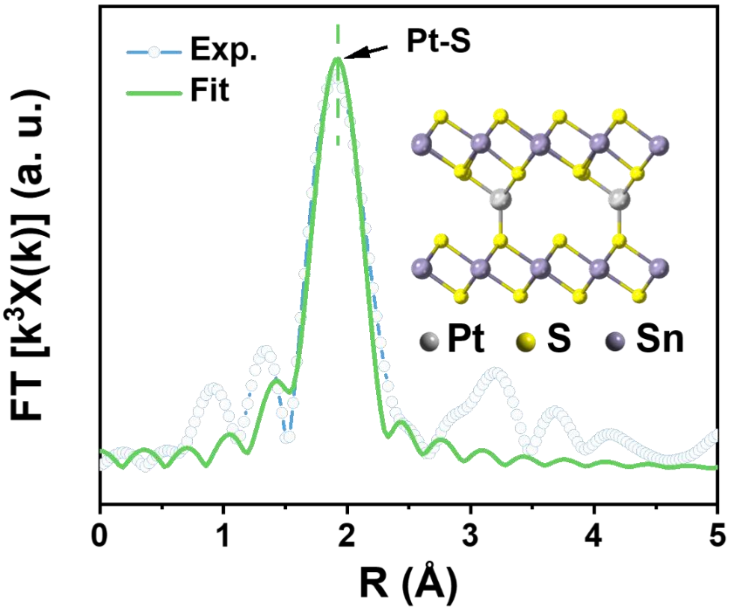

Supplementary Fig. 23 EXAFS fitting. Fourier transform EXAFS (FT-EXAFS) region for the local structure of Pt of Pt-SnS_2/rGO and the corresponding EXAFS fitting curve at R space. The fine fitting illustrates that the single Pt atoms are each coordinated by four S atoms, consistent with the calculated structure of Pt-SnS_2/rGO in the inset.

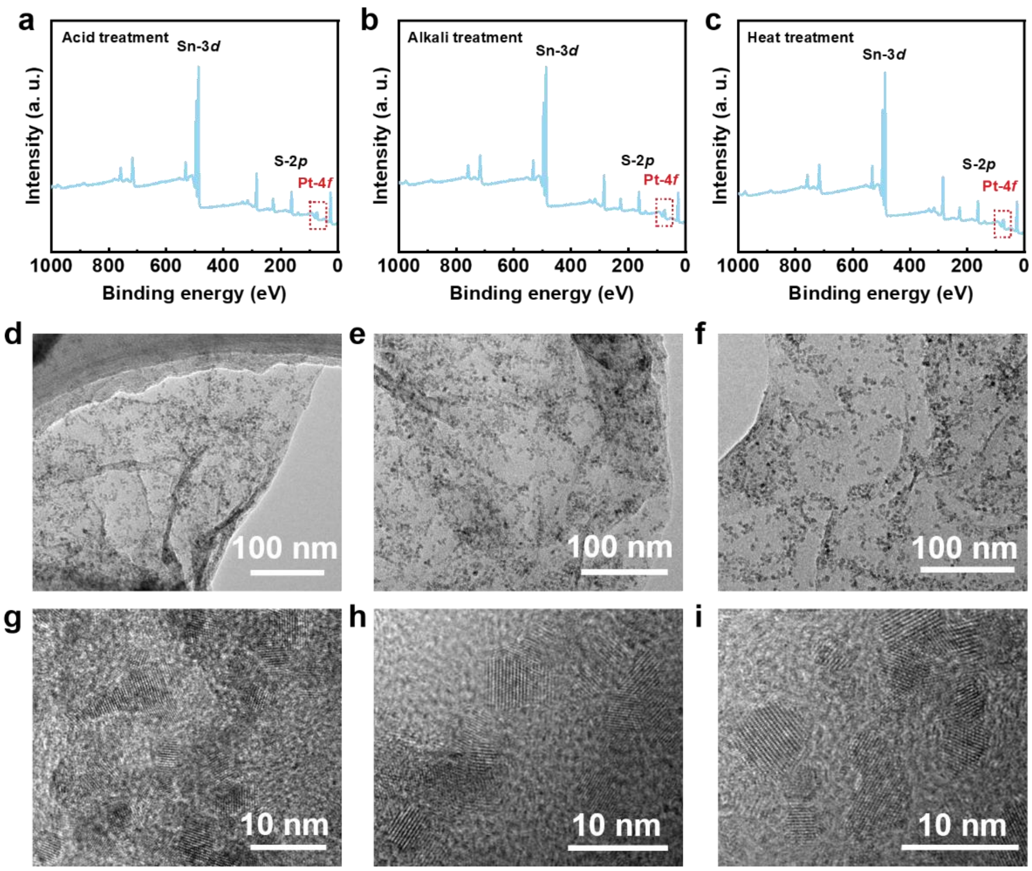

Supplementary Fig. 24 The stability of Pt-SnS_2/rGO. a, XPS for acid treatment in 1 M H_2SO_4 for 5 h. **b,** XPS for alkali treatment in 1 M KOH for 5 h. **c,** XPS for thermal treatment at 100°C in water for 5 h. **d,** The TEM image of Pt-SnS_2/rGO after acid treatment. **e,** The TEM image of Pt-SnS_2/rGO after alkali treatment. **f,** The TEM image of Pt-SnS_2/rGO after thermal treatment. **g,** The HRTEM image of Pt-SnS_2/rGO after acid treatment. **h,** The HRTEM image of Pt-SnS_2/rGO after alkali treatment. **i,** The HRTEM image of Pt-SnS_2/rGO after thermal treatment.

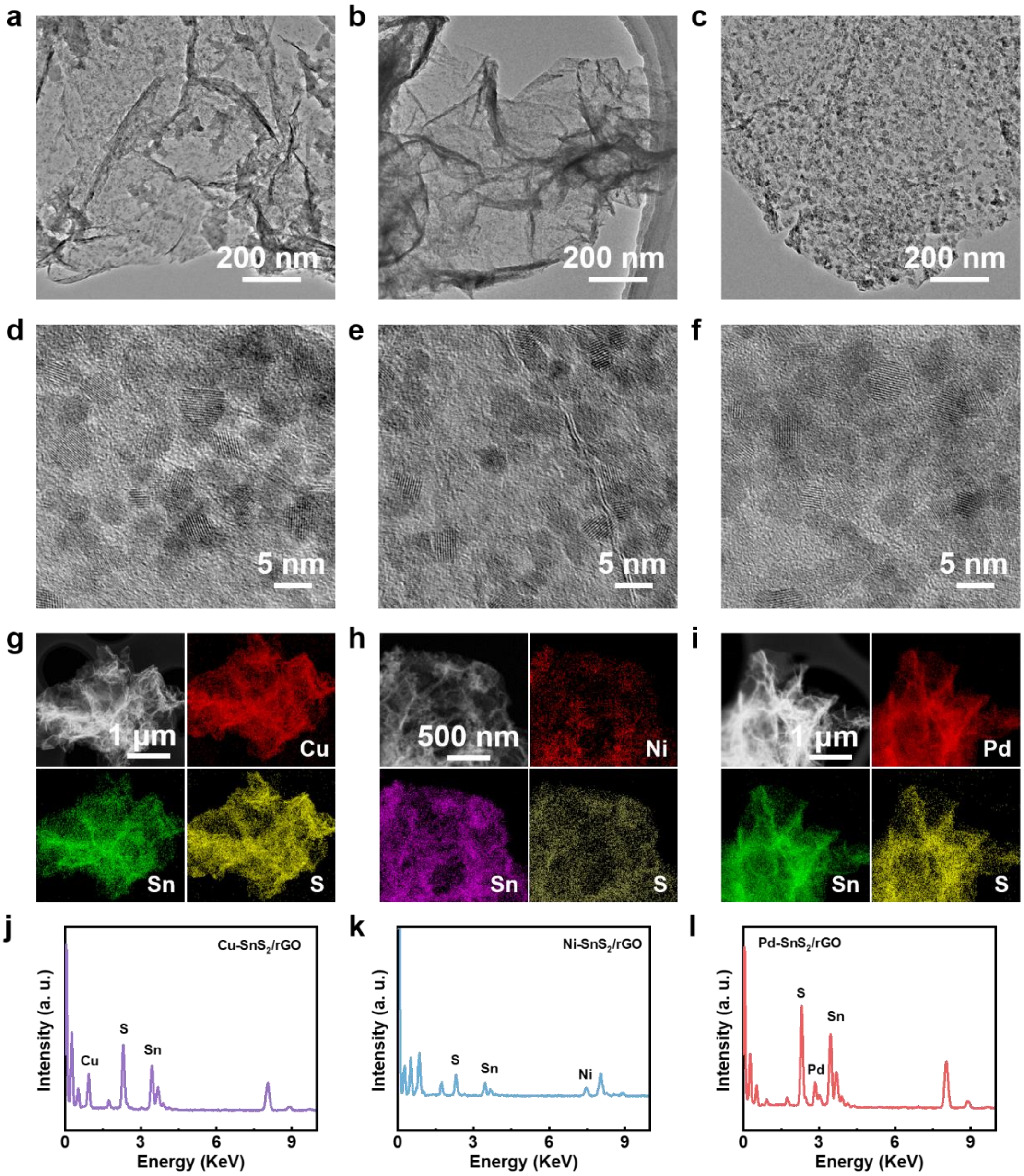

Supplementary Fig. 25 The TEM images of Cu, Ni, and Pd-SnS_2/rGO. a-c, The TEM images of Cu, Ni, and Pd-SnS_2/rGO, respectively. **d-f,** The HRTEM images of Cu, Ni, and Pd-SnS_2/rGO, respectively. **g-i,** The EDS mapping of Cu, Ni, and Pd-SnS_2/rGO, respectively. **j-l,** The EDS spectra of Cu, Ni, and Pd-SnS_2/rGO, respectively.

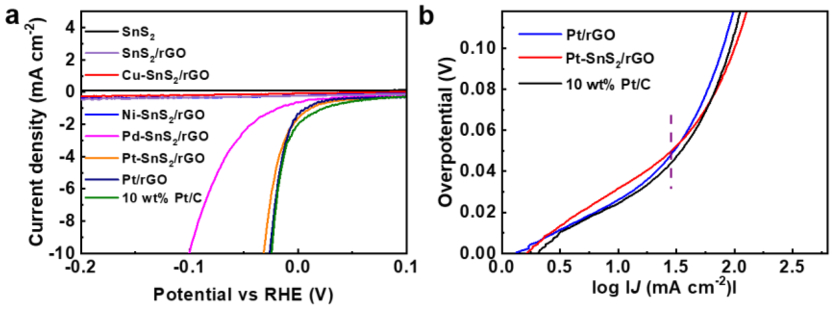

Supplementary Fig. 26 Catalytic performance of the catalysts. a, Magnifying LSV curves of pure SnS_2, pure SnS_2/rGO, Pt-SnS_2/rGO, Pd-SnS_2/rGO, Ni-SnS_2/rGO, Cu-SnS_2/rGO, 10 wt% Pt/rGO and 10 wt% Pt/C. 1 wt% Pt-SnS_2/rGO shows similar overpotentials to 10 wt% Pt/rGO and 10 wt% Pt/C, noticeably better than other intercalated catalysts. **b,** Tafel slopes of 1 wt% Pt-SnS_2/rGO, 10 wt% Pt/rGO, and 10 wt% Pt/C show excellent catalytic activity of Pt-SnS_2/rGO comparable to 10 wt% commercial Pt/C.

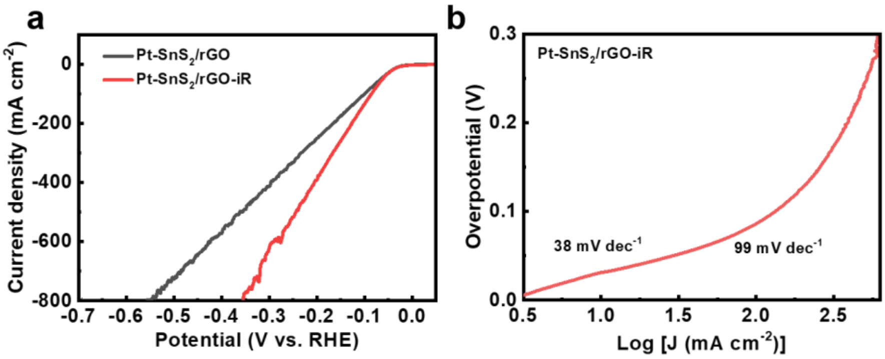

Supplementary Fig. 27 The catalytic data of Pt-SnS_2/rGO after iR compensation. **a,** The LSV curves of Pt-SnS_2/rGO with and without iR compensation. **b,** The tafel curve of Pt-SnS_2/rGO after iR compensation.

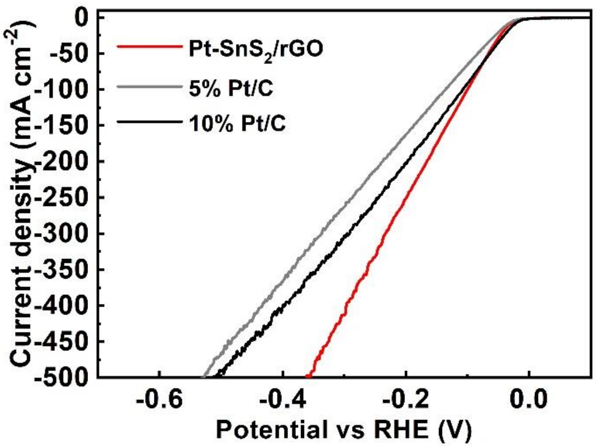

Supplementary Fig. 28 Calculation of the Pt mass activity. To compare the Pt mass activities of Pt-SnS_2/rGO, 5 wt% Pt/C, and 10 wt% Pt/C, their activity values have been normalized to Pt loadings. The overpotential of -0.4 V was selected to evaluate the mass activity. The details are as follows:

$$J_{Mass}^{0.3\%\ Pt-SnS_2/rGO} = \frac{J_{Area}^{0.3\%\ Pt-SnS_2/rGO}\ (mA\ cm^{-2})}{Mass_{Pt}} = \frac{322.85\ (mA\ cm^{-2})}{0.00086\ (mg\ cm^{-2})} = 364.8\text{A mg}^{-1}\ (1)$$

$$J_{Mass}^{1\%\ Pt-SnS_2/rGO} = \frac{J_{Area}^{1\%\ Pt-SnS_2/rGO}\ (mA\ cm^{-2})}{Mass_{Pt}} = \frac{571.98\ (mA\ cm^{-2})}{0.00295\ (mg\ cm^{-2})} = 193.9\text{ A mg}^{-1}\ (2)$$

$$J_{Mass}^{5\%\ Pt/C} = \frac{J_{Area}^{5\%\ Pt/C}\ (mA\ cm^{-2})}{Mass_{Pt}} = \frac{367.87\ (mA\ cm^{-2})}{0.01405\ (mg\ cm^{-2})} = 26.2\text{ A mg}^{-1}\ (3)$$

$$J_{Mass}^{10\%\ Pt/C} = \frac{J_{Area}^{10\%\ Pt/C}\ (mA\ cm^{-2})}{Mass_{Pt}} = \frac{403.96\ (mA\ cm^{-2})}{0.02810\ (mg\ cm^{-2})} = 14.4\text{ A mg}^{-1}\ (4)$$

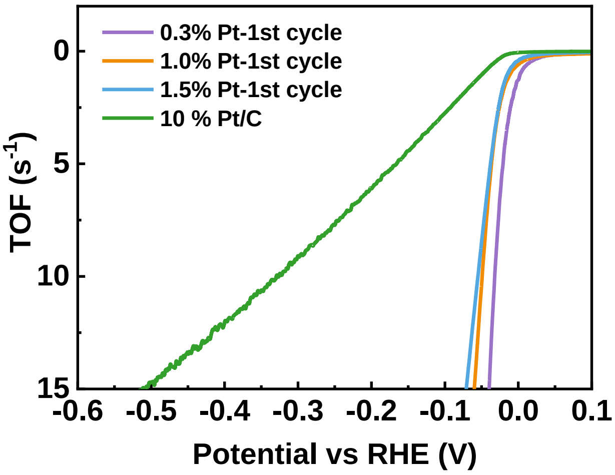

Supplementary Fig. 29 The comparison of TOF curves. TOF curves of Pt-SnS_2/rGO with different Pt contents and 10 wt% Pt/C. The Pt contents are 0.3 wt%, 1.0 wt%, and 1.5 wt%, respectively.

Calculation of the TOF and number of active sites: The TOF value per Pt site was calculated by the following equation:

$$\text{TOF} = \frac{\text{Total hydrogen turnovers per geometric area}}{\text{Active sites per geometic area}} \; (5)$$

The total hydrogen turnover was calculated from the current density:

Total hydrogen turnovers =

$$\left(j\,\frac{\text{mA}}{\text{cm}^2}\right)\left(\frac{1\ \text{C s}^{-1}}{1000\ \text{mA}}\right)\left(\frac{1\ \text{mol e}^-}{96485.3\ \text{C}}\right)\left(\frac{1\ \text{mol H}_2}{2\ \text{mol e}^-}\right)\left(\frac{6.022\ \times\ 10^{23}}{1\text{mol H}_2}\right)$$

$$= 3.12\ \times\ 10^{15}\ \frac{H_2\ \text{s}^{-1}}{\text{cm}^2}/(\text{mA cm}^{-2})\ (6)$$

The Pt content in Pt-SnS2/rGO was calculated to be 0.3 wt%, 1.0 wt%, and 1.5 wt% from the ICP-MS data.

For Pt0.3-SnS2/rGO,

Active sites (Pt) =

$$\left(\frac{\text{Pt wt\%}\ \times\ \text{catalyst loading per geometric area (g/cm}^2)}{M_{w_{\text{Pt}}}\ \text{(g/mol)}}\right)\left(\frac{6.022\ \times\ 10^{23}}{1\ \text{mol Pt}}\right)$$

$$= \left(\frac{0.3\%\times 2.83\times 10^{-4}\ (\text{g/cm}^2)}{195.05\ \text{g/mol}}\right)\left(\frac{6.022\times 10^{23}}{1\ \text{mol Pt}}\right) = 2.62\ \times\ 10^{15}\ \text{cm}^{-2}\ (7)$$

Finally, the current density from the LSV polarisation curve was converted to the TOF value by

$$\text{TOF}_{\text{Pt/(Pt}_{0.3}\text{-SnS}_2\text{/rGO)}} = \left(\frac{3.12\times 10^{15}}{2.62\times 10^{15}}\ \times\ j\right) = 1.19j\ (8)$$

The platinum contents of Pt1.0-SnS2/rGO and Pt1.5-SnS2/rGO were approximately 1.0 wt% and 1.5 wt%, respectively. Therefore,

$$TOF_{Pt/(Pt_{1.0}\text{-}SnS_2/rGO)} = (\frac{3.12 \times 10^{15}}{8.74 \times 10^{15}} \times j) = 0.36j \ (9)$$

$$TOF_{Pt/(Pt_{1.5}\text{-}SnS_2/rGO)} = (\frac{3.12 \times 10^{15}}{13.1 \times 10^{15}} \times j) = 0.24j \ (10)$$

$$TOF_{Pt/(10\ wt\%\ Pt/C)} = (\frac{3.12 \times 10^{15}}{8.73 \times 10^{16}} \times j) = 0.03j \ (11)$$

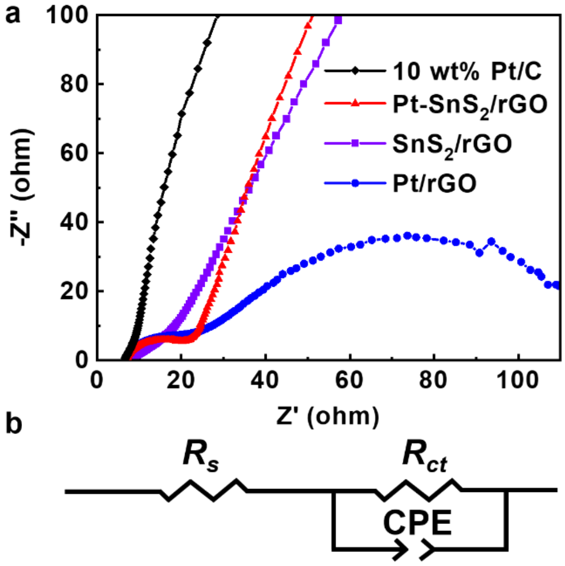

Supplementary Fig. 30 EIS data. a, Nyquist plots of 1 wt% Pt-SnS_2/rGO, SnS_2/rGO, 10 wt% Pt/rGO, and 10 wt% Pt/C at 50 mV vs. RHE. The reduced impedance during electron transport of 1 wt% Pt-SnS_2/rGO is confirmed. **b,** The corresponding equivalent circuit.

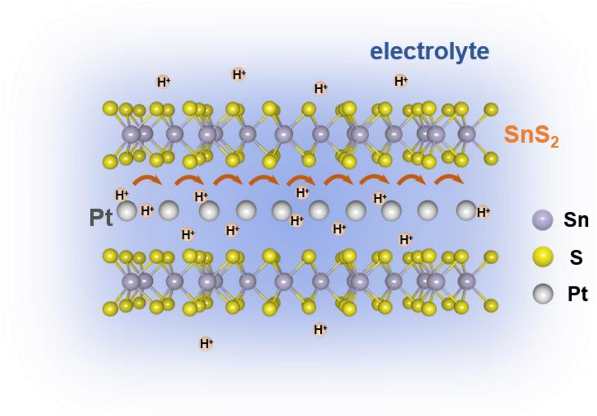

Supplementary Fig. 31 The schematic diagram of Pt-intercalated SnS_2 in the electrolyte. The Pt atoms are located in the vdW gap of SnS_2 and H^+ can migrate in the vdW gap.

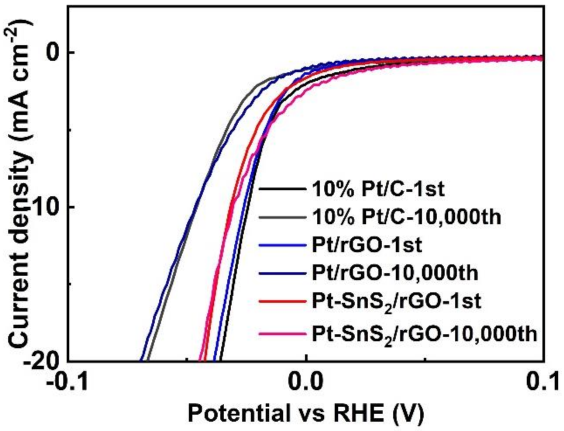

Supplementary Fig. 32 Magnifying LSV curves of 1 wt% Pt-SnS_2/rGO, Pt/rGO, and 10 wt% Pt/C before and after cycles. The 1 wt% Pt-SnS_2/rGO sample exhibits the most changeless overpotential even after 50,000 cycles compared to the 10 wt% Pt/rGO and 10 wt% Pt/C after just 10,000 cycles.

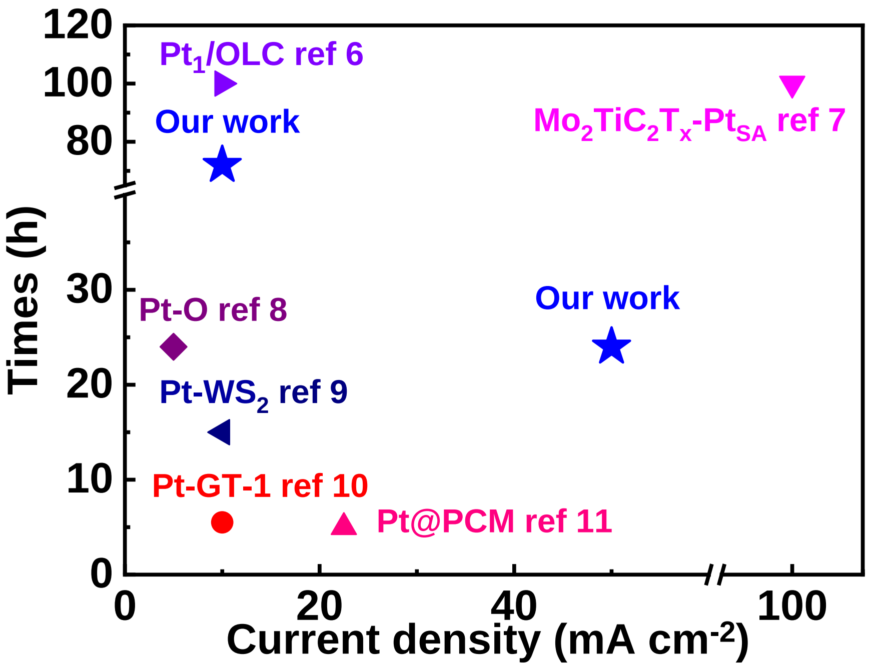

Supplementary Fig. 33 Comparison of stability. The comparison of different current densities and corresponding cycle time in the i-t test between 1 wt% Pt-SnS_2/rGO and other materials reported in the literature. Our catalyst is superior to most Pt-based HER catalysts reported before.

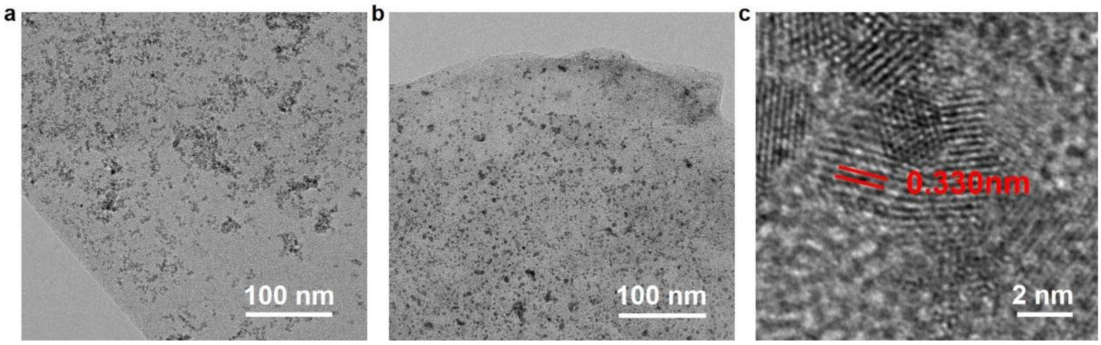

Supplementary Fig. 34 Structure stability during cycling. a, The TEM image of Pt-SnS_2/rGO before cycling. **b,** The TEM image of Pt-SnS_2/rGO after 50,000 cycles. The unchangeable morphology suggests good stability of Pt-SnS_2/rGO during cycling. **c,** The HRTEM image of Pt-SnS_2/rGO after 50,000 cycles shows a stable lattice of intercalated SnS_2.

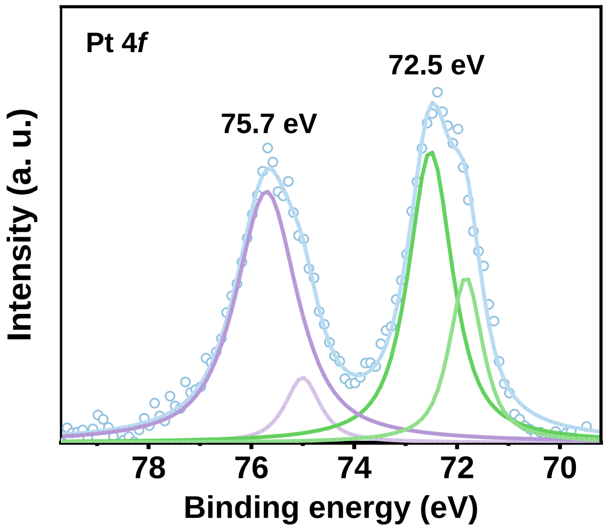

Supplementary Fig. 35 The XPS spectrum of Pt 4*f* of Pt-SnS_2/rGO after 50,000 cycles. The two peaks are approximate to those of the sample before cycling, indicating prominent structure stability of this catalyst during the cycling process.

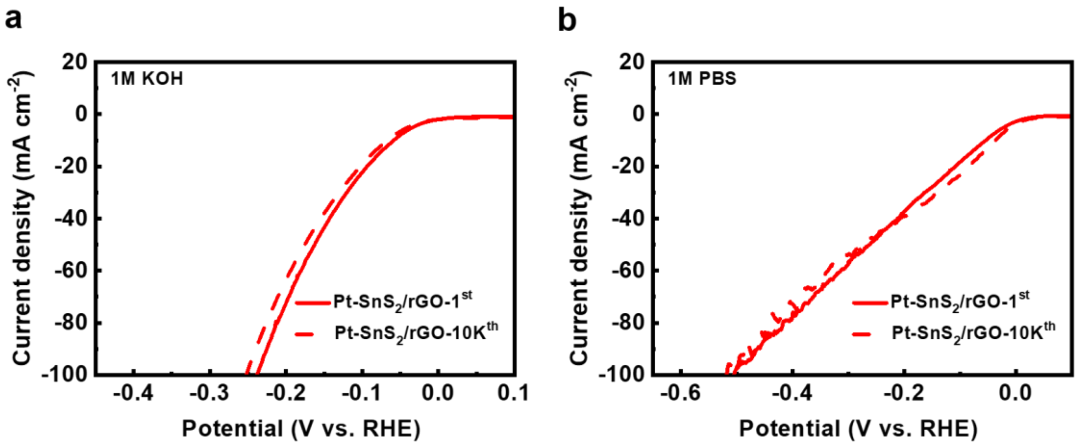

Supplementary Fig. 36 HER data in alkaline and neutral conditions. a, The LSV curves of Pt-SnS_2/rGO in 1 M KOH before and after 10,000 cycles. **b,** The LSV curves of Pt-SnS_2/rGO in 1 M PBS before and after 10,000 cycles.

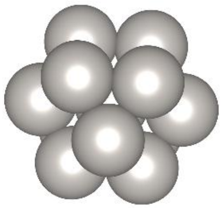

Supplementary Fig. 37 Structure of magic-number Pt_{13} cluster. Pt_{13} is a reference for formation energy calculation of Pt atoms. The Pt-Pt bond length of Pt_{13} is 2.67Å.

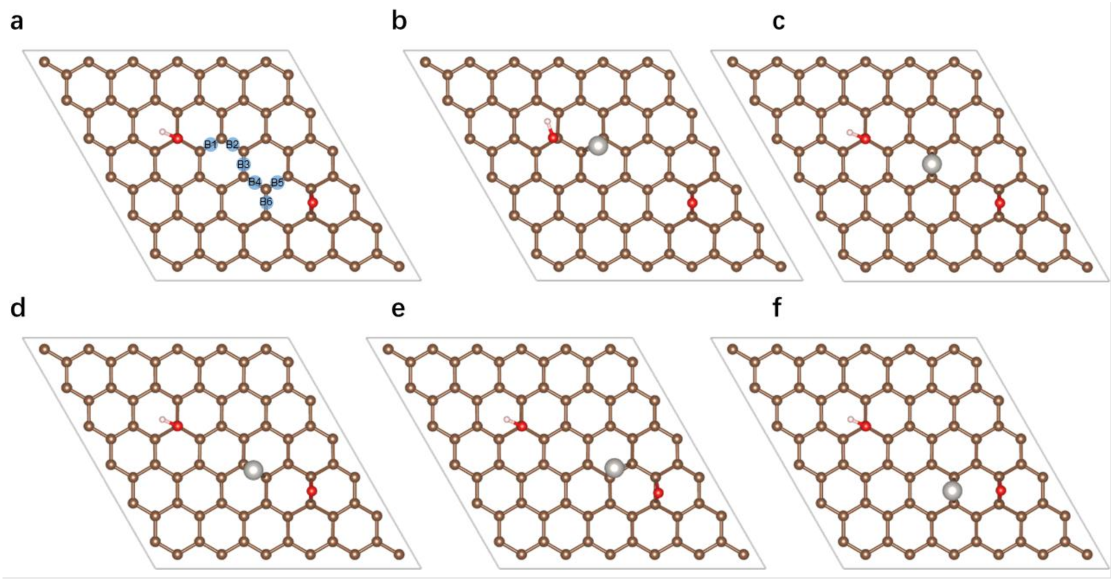

Supplementary Fig. 38 Atomic configurations for SA Pt adsorbed on the rGO surface. a, Since Pt adsorption at the C-C bridge site is most favorable, six bridge adsorption sites on the rGO surface are considered. **b-f,** Optimized configurations for SA Pt adsorbed B1(B2), B3, B4, B5, B6 sites. For B2 adsorption configuration, SA Pt moves into B1 site upon structural relaxation.

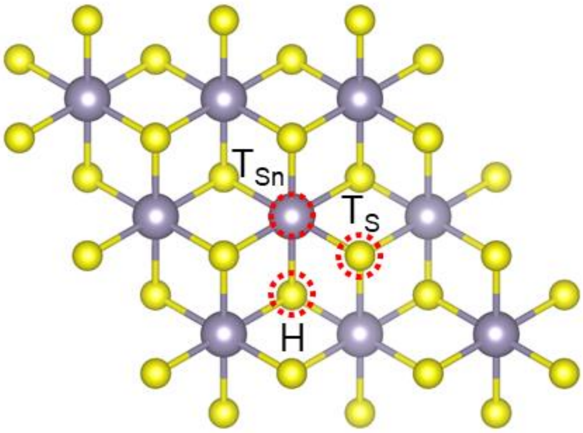

Supplementary Fig. 39 Three considered adsorption sites for SA Pt atom on top of SnS_2 surface, i.e., on top of the Sn or S site (T_{Sn} or T_S), the hollow site (H). The thermodynamic stabilities of different adsorption systems are accessed by their formation energies, calculated as $E_f=E_{tot}-E_{ref}-\mu_{Pt}$[4], where E_{tot} and E_{ref} are the total energies of SnS_2 systems with and without Pt adsorption, respectively. The μ_{Pt} is the chemical potential of Pt in the magic-number Pt_{13} cluster with the structure shown in **Supplementary Fig. 37.** The calculated formation energies are -1.34, -0.32, and 0.85 eV for Pt adsorption on T_{Sn}, H, and T_S sites, respectively.

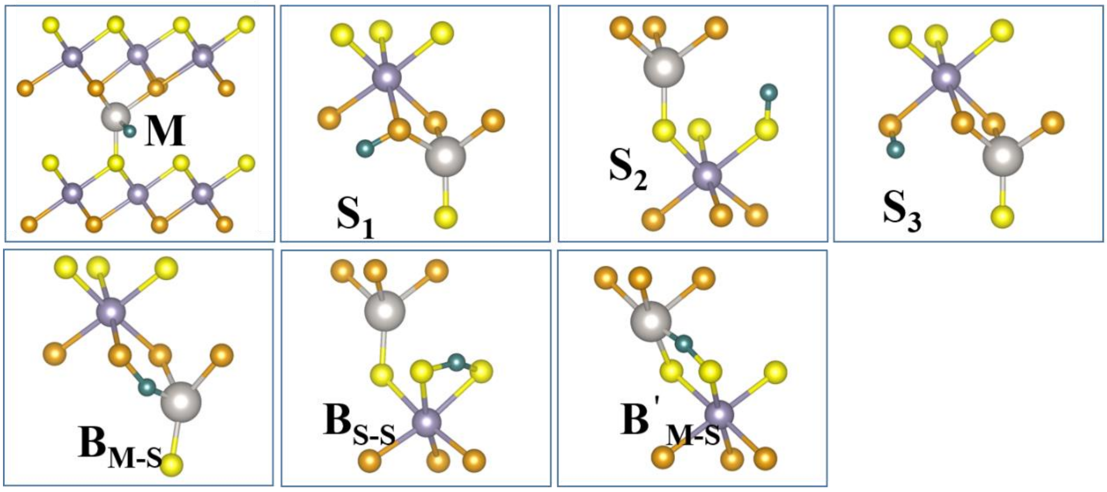

Supplementary Fig. 40 The possible active sites calculated for hydrogen adsorption of metal-intercalated SnS_2. The active sites include M, S_1, S_2, S_3, $B_{M\text{-}S}$, $B_{S\text{-}S}$ and $B'_{M\text{-}S}$, respectively. $B_{M\text{-}S}$, $B_{S\text{-}S}$ and $B'_{M\text{-}S}$ represent three kinds of bridge structures for hydrogen adsorption while the sites M, S_1, S_2, S_3 refer to direct adsorption of hydrogen to the individual sites.

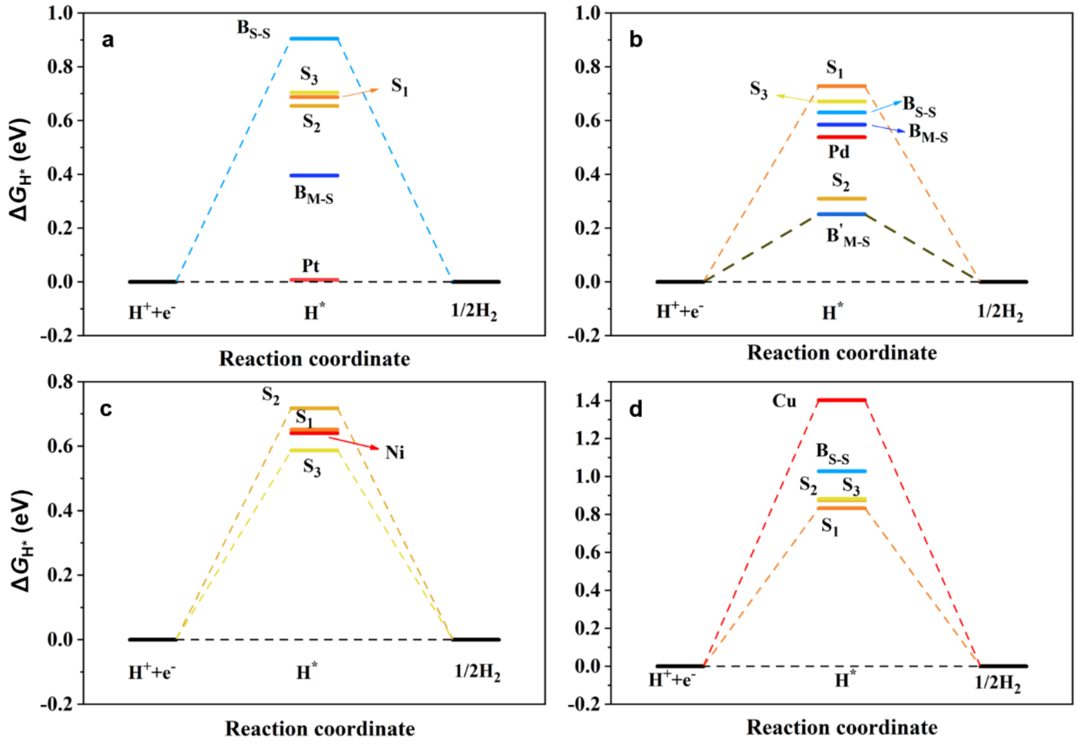

Supplementary Fig. 41 The different |ΔG_{H*}| values of the active sites of metal-intercalated SnS_2. a, Pt-SnS_2. **b,** Pd-SnS_2. **c,** Ni-SnS_2. **d,** Cu-SnS_2. The Pt site in Pt-SnS_2 presents the lowest value of |ΔG_{H*}| (0.01 eV), while B'$_{M-S}$ (|ΔG_{H*}| is 0.25 eV), S_3 (|ΔG_{H*}| is 0.59 eV), and S_1 (|ΔG_{H*}| is 0.83 eV) sites are the most active sites for Pd-, Ni- and Cu-SnS_2, respectively.

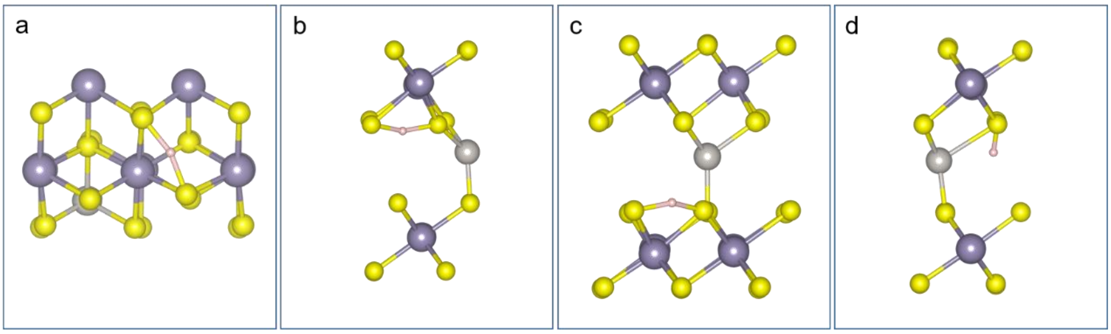

Supplementary Fig. 42 The transitional state (TS) of different H-migration pathways for Pt-SnS_2. a, The TS of S to S(1) pathway. **b,** The TS of S to S(2) pathway. **c,** The TS of S to S(3) pathway. **d,** The TS of S to Pt pathway.

Supplementary Tables

Supplementary Table. 1 | The interlayer spacing of NbS_2, TaS_2 and corresponding intercalated materials

	d_c (Å)		d_c (Å)
NbS_2	5.98	TaS_2	6.01
$Mn_{1/2}NbS_2$	6.25	$Mn_{1/3}TaS_2$	6.34
$Mn_{1/3}NbS_2$	6.31	$Fe_{1/3}TaS_2$	6.14
$Mn_{1/4}NbS_2$	6.22	$Co_{1/3}TaS_2$	5.96
$Fe_{1/3}NbS_2$	6.10	$Ni_{1/3}TaS_2$	5.96
$Co_{1/3}NbS_2$	5.96		
$Ni_{1/3}NbS_2$	5.94		
$Nb_{1.06}S_2$	5.97		

Supplementary Table. 2 | Comparison of i-t performance between Pt-SnS_2/rGO and other Pt based materials reported in the literature.

Material	Current density ($mA\ cm^{-2}$)	Times (h)
Pt-SnS_2/rGO (Our work)	10	72
Pt-SnS_2/rGO (Our work)	50	24
Pt1/OLC ref 6	10	100
$Mo_2TiC_2T_x$-Pt_{SA} ref 7	100	100
Pt-O ref 8	5	24
Pt-WS_2 ref 9	10	15
Pt-GT-1 ref 10	~10	5
Pt@PCM ref 11	~22	5

Supplementary Table. 3 | The comparison between our Pt-SnS_2/rGO and other Pt single atoms (Pt SA)[11], Pt-Ru alloy[12]and low Pt[9] reported in the literature, the potential increase per cycle under various current density.

Material	Cycle numbers	Current density ($mA\ cm^{-2}$)	Potential increase /cycle (μA)
Pt-SnS_2/rGO (Our work)	50,000	100	0.1
Pt SA ref 12	10,000	60	0.4
Pt-Ru ref 13	5,000	~30	0.5
Low Pt ref 10	10,000	~10	2.8
10% Pt/C	10,000	100	9.5

Supplementary Table. 4 | The formation energies of various favorable single-atom Pt adsorption sites on the rGO surface

Adsorption sites	Formation energy (eV)
B1	1.74

B3	1.94
B4	1.83
B5	1.77
B6	1.97

Supplementary References

1. Kresse, G., Furthmüller, J. Efficient iterative schemes for ab initio total-energy calculations using a plane-wave basis set. *Phy. Rev. B* **54**, 11169-11186 (1996).
2. Bučko, T., Lebègue, S., Hafner, J., Ángyán, J. G. Tkatchenko-Scheffler van der Waals correction method with and without self-consistent screening applied to solids. *Phy. Rev. B* **87**, 064110 (2013).
3. Zou, X., Liu, M., Shi, Z. & Yakobson, B. I. Environment-Controlled Dislocation Migration and Superplasticity in Monolayer MoS_2. *Nano Lett.* **15**, 3495–3500 (2015).
4. Zou, X. & Yakobson, B. I. Metallic High-Angle Grain Boundaries in Monolayer Polycrystalline WS2. *Small* **11**, 4503–4507 (2015).
5. Zou, X. L., Wang, L. Q., Yakobson, B. I. *Nanoscale,* **10**, 1129-1134 (2018).
6. Liu, D. et al. Atomically dispersed platinum supported on curved carbon supports for efficient electrocatalytic hydrogen evolution. *Nat. Energy* **4,** 512-518 (2019).
7. Zhang, J. Q. et al. Single platinum atoms immobilized on an MXene as an efficient catalyst for the hydrogen evolution reaction. *Nat. Catal.* **1**, 985–992 (2018).
8. Yu, F. L. et al. Pt-O bond as an active site superior to Pt^0 in hydrogen evolution reaction. *Nat. Commun.* **11,** 490-496 (2020).
9. Tang, K. et al. High Edge Selectivity of In Situ Electrochemical Pt Deposition on Edge-Rich Layered WS_2 Nanosheets. *Adv. Mater.* **30**, 1704779-1704785 (2018).
10. Tiwari, J. N. et al. Multicomponent electrocatalyst with ultralow Pt loading and high hydrogen evolution activity. *Nat. Energy* **3**, 773–782 (2018).
11. Zhang, H. B. et al. Dynamic traction of lattice-confined platinum atoms into mesoporous carbon matrix for hydrogen evolution reaction. *Sci. Adv.* **4**, eaao6657-eaao6665 (2018).
12. Cheng, N. C. et al. Platinum single-atom and cluster catalysis of the hydrogen evolution reaction. *Nat. Commun.* **7,** 13638-13646 (2016).
13. Zhang, L. et al. Atomic layer deposited Pt-Ru dual-metal dimers and identifying their active sites for hydrogen evolution reaction. *Nat. Commun.* **10,** 4936-4946 (2019).
